# Long Noncoding RNA MALAT1 Controls Cell Cycle Progression by Regulating the Expression of Oncogenic Transcription Factor B-MYB

**DOI:** 10.1371/journal.pgen.1003368

**Published:** 2013-03-21

**Authors:** Vidisha Tripathi, Zhen Shen, Arindam Chakraborty, Sumanprava Giri, Susan M. Freier, Xiaolin Wu, Yongqing Zhang, Myriam Gorospe, Supriya G. Prasanth, Ashish Lal, Kannanganattu V. Prasanth

**Affiliations:** 1 Department of Cell and Developmental Biology, University of Illinois at Urbana-Champaign, Urbana, Illinois, United States of America; 2 ISIS Pharmaceuticals, Carlsbad, California, United States of America; 3 Laboratory of Molecular Technology, SAIC-Frederick, Frederick National Laboratory for Cancer Research, Frederick, Maryland, United States of America; 4 Research Resources Branch, National Institute on Aging, National Institutes of Health, Baltimore, Maryland, United States of America; 5 Laboratory of Molecular Biology and Immunology, National Institute of Aging, National Institutes of Health, Baltimore, Maryland, United States of America; 6 Genetics Branch, National Cancer Institute, National Institutes of Health, Bethesda, Maryland, United States of America; Fred Hutchinson Cancer Research Center, United States of America

## Abstract

The long noncoding MALAT1 RNA is upregulated in cancer tissues and its elevated expression is associated with hyper-proliferation, but the underlying mechanism is poorly understood. We demonstrate that MALAT1 levels are regulated during normal cell cycle progression. Genome-wide transcriptome analyses in normal human diploid fibroblasts reveal that MALAT1 modulates the expression of cell cycle genes and is required for G1/S and mitotic progression. Depletion of MALAT1 leads to activation of p53 and its target genes. The cell cycle defects observed in MALAT1-depleted cells are sensitive to p53 levels, indicating that p53 is a major downstream mediator of MALAT1 activity. Furthermore, MALAT1-depleted cells display reduced expression of *B-MYB (Mybl2)*, an oncogenic transcription factor involved in G2/M progression, due to altered binding of splicing factors on B-MYB pre-mRNA and aberrant alternative splicing. In human cells, MALAT1 promotes cellular proliferation by modulating the expression and/or pre-mRNA processing of cell cycle–regulated transcription factors. These findings provide mechanistic insights on the role of MALAT1 in regulating cellular proliferation.

## Author Summary

The mammalian genome encodes large number of long non protein-coding RNAs (lncRNAs). These lncRNAs are suggested to regulate key biological processes (including cellular proliferation and differentiation), and aberrant expression of these is associated with cancer. However, only a few of these lncRNAs have been functionally validated in biological or disease processes. MALAT1, an abundant nuclear-retained lncRNA, is overexpressed in several cancers, and its elevated expression has been associated with hyper-proliferation and metastasis. However, the underlying mechanism behind this deregulation and its association with cancer is poorly understood. Here, we establish the role of MALAT1 in the cell cycle pathway and propose the molecular mechanism of its function during normal cell cycle progression. MALAT1 RNA levels are differentially regulated and critical for normal cell cycle progression. Depletion of MALAT1 results in cell cycle arrest with significantly reduced cellular proliferation, simultaneously leading to activation of p53 and its target genes. Further, the accurate levels of MALAT1 in the cell are extremely crucial for expression and activity of the oncogenic transcription factor B-MYB, which is involved in G2/M progression. Our data indicates that the cancer-associated MALAT1 RNA regulates cellular proliferation by modulating the expression and/or pre-mRNA processing of cell cycle–regulated transcription factors.

## Introduction

The eukaryotic genome harbors a large number of noncoding RNAs, which include small and long noncoding RNAs (lncRNAs) [1], [2], [3], [4]. Small ncRNAs such as microRNAs regulate the expression of target genes at the level of translation or mRNA stability, whereas piwi-interacting RNAs (piRNA) have been linked to transcriptional gene silencing of retrotransposons and other repeat-containing genetic elements [5], [6], [7], [8]. In addition to the class of well-studied small ncRNAs, lncRNAs, which are noncoding transcripts that are >200 nucleotides in length, have recently emerged as important molecules in several cellular processes [4], [9], [10]. The human genome encodes ∼15,000–17,000 potential lncRNAs. However, the function of less than 2% of the human lncRNAs is clearly elucidated [11], [12]. LncRNAs are involved in several crucial functions: they can act as a scaffold to keep several proteins tethered to a specific cellular compartment, act as a guide to recruit proteins to a specific chromatin site or influence local chromatin architecture [3], [4], [13], [14], [15]. For instance, the X-chromosome encoded Xist lncRNA coats the inactive X-chromosome (Xi) in female mammals and facilitates the recruitment of chromatin modifiers to the Xi [16], [17]. Similarly, the imprinted Air lncRNA interacts with the histone-methyl transferase G9a and recruits it to epigenetically silence *Slc22a3* [18], [19], [20]. Further, nuclear-retained lncRNAs act as structural components of specific sub-nuclear domains [21], [22], [23], [24]. LncRNAs are also known to regulate transcription and RNA processing events and also serve as precursors for small RNAs [14], [15].

Several lncRNAs exhibit temporal and spatial expression patterns or their expression is restricted to particular tissue or cell types or cell cycle stages, indicating vital and diverse biological roles of lncRNAs [4], [25], [26], [27], [28]. A recent study demonstrated cell cycle-regulated expression of several of the lncRNAs in mammalian cells [29]. The authors identified >200 lncRNAs encoded in close proximity to more than 50 protein-coding genes involved in cell cycle, including cyclins and cyclin-dependent kinases. During cell cycle progression, the levels of both lncRNAs and the nearby cell cycle gene mRNAs displayed dynamic fluctuations. However, the expression of these lncRNAs did not correlate either positively or negatively with the expression of the nearby cell cycle genes [29]. This implies that although some lncRNAs and mRNAs could be regulated in concert, they may not necessarily regulate each other. On the other hand, several other studies have demonstrated the involvement of lncRNAs in regulating the expression of cell cycle genes in *cis* [30], [31], [32], [33]. The ANRIL lncRNA is located upstream of the tumor suppressor locus encoding *p16^INK4A^* and *p15^INK4B^*, mutations or depletion of ANRIL results in the loss of *p16^INK4A^* and *p15^INK4B^* repression [30], [31], [32]. Similarly, an lncRNA transcribed from the 5′regulatory region of *Cyclin D1* (*CCND1*) recruits TLS, an RNA-binding protein, to the *CCND1* gene in response to DNA damage, and results in the transcription repression of *CCND1* [33].

A large number of lncRNAs display deregulated expression in human cancer samples and are regulated by oncogenic or tumor suppressor pathways [28], [34], [35], [36], [37], [38]. The HOTAIR lncRNA, which is known to regulate the expression of HOX gene clusters, is highly induced in breast cancer samples and its elevated expression has been correlated with metastasis and death [39]. Recent studies have also demonstrated the involvement of lncRNAs in the p53 gene regulatory pathway [4]. For example, lincRNA-p21 is activated by p53 and serves as a repressor in the p53-dependent transcriptional network [40]. DNA damage induces the expression of another lncRNA, PANDA in a p53-dependent manner. PANDA is transcribed from the *p21* (*CDKN1A*) promoter, and it negatively regulates the expression of pro-apoptotic genes upon DNA damage, thereby controlling apoptosis [29]. Finally MEG3, an imprinted lncRNA induces accumulation of p53 by negatively regulating MDM2 expression [41].

The lncRNA MALAT1 is upregulated in several solid tumors and its differential expression is linked with cancer metastasis and recurrence [15], [28], [42]. MALAT1 is a highly abundant nucleus-restricted RNA that localizes to nuclear speckles, a sub-nuclear domain suggested to coordinate RNA polymerase II transcription, pre-mRNA splicing and mRNA export [43], [44], [45]. MALAT1 interacts with several pre-mRNA splicing factors including serine arginine dipeptide-containing SR family splicing factors [46], [47], [48], [49], [50], [51]. Furthermore, MALAT1 modulates the cellular distribution and activity of SR splicing factors thereby influencing alternative splicing of pre-mRNAs [50]. By utilizing such a mechanism, cells could alter the local concentration of a particular splicing factor upon a specific external signal or during specific stages of the cell cycle.

Previous studies have shown that transient overexpression of MALAT1 enhanced cellular proliferation in cell lines and tumor formation in nude mice, while depletion of MALAT1 in tumor cells reduced tumorigenicity [52], [53]. A recent study suggested the involvement of MALAT1 in regulating the E2F1 transcription factor activity, which is a crucial determinant of cell cycle progression and tumorigenesis [54]. These results indicate that MALAT1 has a pro-proliferative function; however, the mechanism has yet to be identified. In the present study, we examined the role of MALAT1 in cell cycle progression. We demonstrate that in human cells, MALAT1 levels are regulated during the cell division cycle. The differential levels of MALAT1 during specific cell cycle stages influence the expression of genes involved in cell cycle progression. Furthermore, MALAT1 depletion in normal human diploid fibroblasts (HDFs) induces DNA-damage response and results in the activation of p53 and its target genes. The cell cycle defects observed in MALAT1-depleted cells are sensitive to the cellular levels of p53, indicating that p53 is an important effector of MALAT1 function. Finally, we establish that the pro-proliferative role of MALAT1 is accomplished by its involvement in regulating the expression and/or pre-mRNA processing of oncogenic transcription factors, especially those that control mitotic progression.

## Results

### MALAT1 controls cell cycle progression in human cells

**Figure 1:**
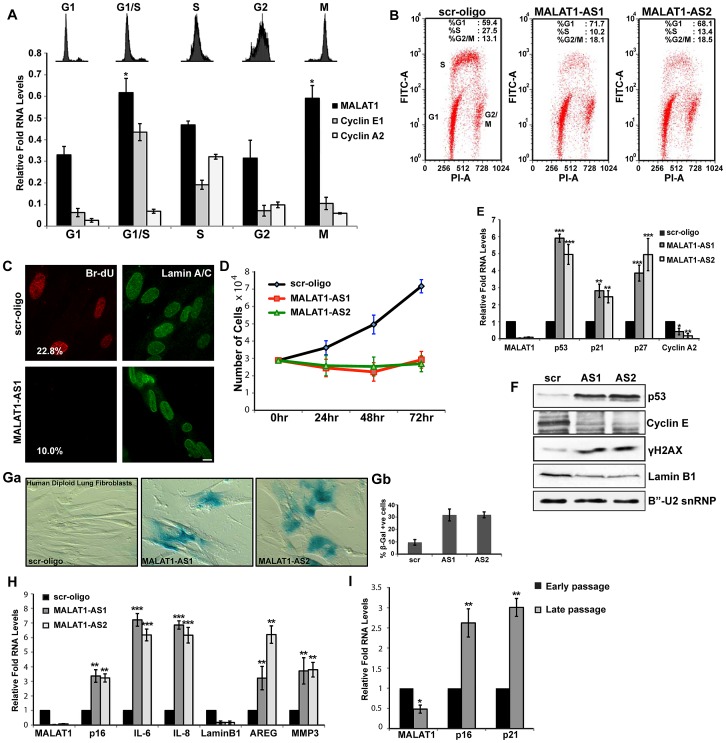
MALAT1 levels are cell cycle regulated and depletion of MALAT1 results in proliferation defect. (A) Flow cytometry and qRT-PCR analyses on cell cycle-synchronized U2OS cells. (B) BrdU-PI flow cytometry analyses in control (scr-oligo) and MALAT1-depleted (MALAT1-AS1 & -AS2) human diploid lung fibroblasts (WI-38). (C) BrdU pulse-labeling (red) and lamin A/C immunostaining (green) in control and MALAT1-depleted WI-38 cells. The number designates the percentage of BrdU-incorporating cells. (D) Proliferation assay shows reduced proliferation in MALAT1-depleted WI-38 cells. (E–F) The expression of indicated genes is determined by qRT-PCR and immunoblot assays from control and MALAT1-depleted WI-38 cells. (Ga–b) β-galactosidase staining in control and MALAT1-depleted WI-38 cells. Gb denotes the percentage of β-gal +ve cells. (H) The relative expression of indicated senescence-associated genes is determined by qPCR from control and MALAT1-depleted WI-38. (I) The relative expression of indicated genes is assessed by qRT-PCR from proliferating (early passage) and replicative senescent (late passage) WI-38 cells. Note that replicative senescent cells show reduced levels of MALAT1. Mean ± SEM, *p<0.05, **p<0.01 and ***p<0.001.

In order to understand the role of MALAT1 in cellular proliferation, we synchronized human osteosarcoma cells (U2OS) in specific cell cycle stages (Figure 1A) and examined the levels of MALAT1 in each stage of the cell cycle. Quantitative RT-PCR (qRT-PCR) results revealed cell cycle-dependent expression of MALAT1, with low levels during G1 and G2 and high levels during G1/S and mitosis (M) (Figure 1A). Similar cell cycle regulation of MALAT1 was also observed in WI-38 human diploid lung fibroblasts (HDFs; Figure S1A). We have previously proposed that MALAT1 modulates pre-mRNA splicing by titrating the cellular levels of SR splicing factors [15], [50]. This prompted us to also examine the levels of SR proteins during various phases of cell cycle. SRSF1 levels remained unaltered during cell cycle (Figure S1B), indicating that the changes in MALAT1 levels during the cell cycle could fine tune the association of SR proteins with pre-mRNAs and thereby modulate cell cycle-specific alternative splicing.

To gain insight into the functional relevance of cell cycle-regulated expression of MALAT1, we examined the effects of MALAT1 depletion on cell cycle progression in HDFs that have a finite life span (WI-38 and IMR-90 cells). BrdU-PI and PI flow cytometry data revealed that MALAT1-depleted cells show reduced replication (S-phase) (Figure 1B and Figure S1C). MALAT1-depleted cells also showed an increase in the G1 (scr, 59%: AS1, 72%: AS2, 68%) and a marginal increase in G2/M (scr, 13%: AS1, 18%: AS2, 19%) population (Figure 1B). BrdU pulse labeling as well as proliferation assays confirmed the reduced proliferation of HDFs upon MALAT1 depletion (Figure 1C and 1D). MALAT1-depleted HDFs also showed increased expression of genes that are associated with cell cycle arrest (Figure 1E). The tumor suppressor p53 (TP53) and the cdk inhibitors p21 (a transcriptional target of p53) and p27 were upregulated upon MALAT1 depletion (Figure 1E and 1F). Interestingly, MALAT1-depleted cells also showed increased levels of γH2AX, indicative of double stranded DNA damage response (Figure 1F). Cell cycle arrest phenotypes, including G1 arrest and a significant reduction in S-phase were observed in WI-38 (Figure 1B and Figure S1D) and IMR-90 cells (data not shown) that were depleted of MALAT1 using either multiple independent sets of DNA antisense oligonucleotides or double-stranded siRNAs. These results suggest that MALAT1 is required for cell proliferation of HDFs.

A significant population of the MALAT1-depleted normal fibroblasts showed changes in the cellular morphology with cells appearing flat and displaying ‘fried egg’ morphology, reminiscent of senescent cells (Figure S1E). MALAT1-depleted cells showed enhanced β-galactosidase (β-gal) staining indicative of cellular senescence (Figure 1Ga–b). Furthermore, human fibroblasts that were depleted of MALAT1 (both by DNA antisense oligonucleotides and siRNAs) showed a gene expression signature characteristic of senescent cells (Figure 1H and Figure S1F). Recent studies have demonstrated specific loss of nuclear lamina associated lamin B1 in senescent cells [55], [56]. We also observed reduced lamin B1 mRNA and protein levels in MALAT1-depleted cells (Figure 1F and 1H). Finally, human lung fibroblasts that had undergone replicative senescence showed reduced levels of MALAT1 compared to actively proliferating cells (Figure 1I). Our results demonstrate that MALAT1 levels are cell cycle regulated and depletion of MALAT1 in HDFs results in proliferation defects with a population of cells undergoing senescence.

**Figure 2:**
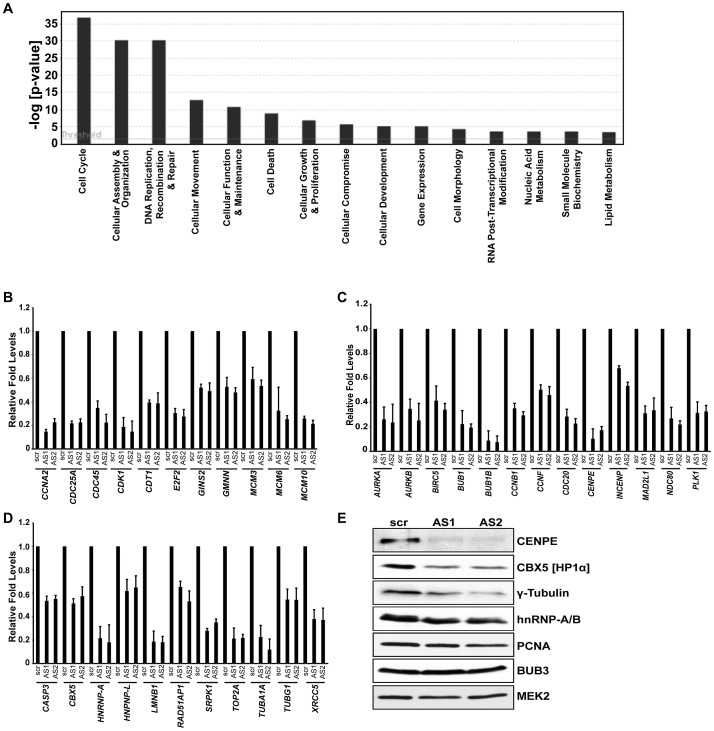
Microarray analysis in HDFs reveal that MALAT1-depletion reduces the expression of genes involved in cell cycle progression. (A) Top significant biological processes for genes whose transcript levels are reduced (Z ratio ≥2.5) in MALAT1-depleted fibroblasts. Cell cycle is the most affected biofunction in MALAT1-depleted cells. (B–D) qRT-PCR analyses in control (scr) versus MALAT1-antisense (AS1 & AS2) oligo-treated HDFs reveal decreased mRNA levels of genes involved in G1/S transition (B), mitotic progression (C), and other general cellular processes (D) upon MALAT1 depletion. (E) The expression of indicated genes is determined by immunoblotting using extracts from control and MALAT1-depleted WI-38 cells. MEK2 is used as loading control.

In order to understand the molecular mechanism underlying the cell cycle arrest upon MALAT1 depletion, we looked at changes in gene expression by performing microarrays from control or MALAT1-depleted HDFs (WI-38) using two independent antisense oligonucleotides (AS1 & AS2) against MALAT1. WI-38 cells were treated with a control (scr) or MALAT1-specific antisense oligonucleotides for 24 hr and then re-transfected with these oligonucleotides for another 24 hr. Total RNA was isolated 24 hr after the second antisense treatment and subjected to microarray analysis. Analyses of the microarray data from triplicate samples revealed that a common set of 413 mRNAs showed ≥2.5-fold reduced abundance in MALAT1-depleted cells (Tables S1, S2, S3). Silencing MALAT1 also increased the expression (≥2.5-fold) of ∼390 genes (Table S4). To gain an insight into the pathways activated by MALAT1, we performed Gene Ontology (GO) analyses of the downregulated genes and found that cell cycle was the most affected biological process in MALAT1-depleted cells (Figure 2A). Using qRT-PCR, we validated the changes in the cellular levels of a large number of mRNAs (∼150) (Figure 2B–2D, Figure S2Aa–c and S2Bc). The genes that were downregulated in MALAT1-depleted cells were broadly classified into several subgroups. A large number of the genes encoding proteins involved in G1/S transition and S-phase progression displayed reduced expression upon silencing of MALAT1 (Figure 2B), corroborating the cell cycle arrest phenotype. The other sub-group of genes, whose expression was severely affected, included those encoding proteins responsible for mitotic progression (Figure 2C). In addition, the expression of genes encoding pre-mRNA processing factors (hnRNPs [hnRNP A, L & K]) and chromatin modifiers (HP1α [CBX5], TOP2A, TOP2B, SMC2 & SMC4) was also compromised in MALAT1-depleted cells (Figure 2D and Figure S2Ab–c). Depletion of MALAT1 using siRNAs also showed similar changes in the expression of genes encoding cell cycle regulatory proteins (Figure S2Ad). Immunoblot analyses further corroborated the qRT-PCR data (Figure 2E). However, the levels of some of the encoded proteins (e.g., BUB3) were comparable between control and MALAT1-depleted cells, even though mRNA levels were somewhat reduced in MALAT1-depleted cells (Figure 2E and Figure S2Aa). Based on our microarray results, it was evident that several E2F target mRNAs were downregulated upon MALAT1 depletion. In this regard, a recent study documented the role for MALAT1 in controlling E2F1 function [54]. Surprisingly, a few of the bona fide E2F1 target gene mRNA levels remained unaltered in MALAT1-depleted cells (PCNA, Figure S2Ae), suggesting that MALAT1 downregulation in fibroblasts did not completely disrupt E2F1 activity.

GO analyses indicated that the p53 signaling pathway was the second most represented biofunctional pathway that was activated upon MALAT1 depletion (Figure S2Bb). This was further confirmed by the observation that MALAT1-depleted cells showed increased expression of p53 and several of its target genes (p21 [CDKN1A], GADD45A, 45B, TP53INP1; Figure 1E, 1F and Figure S2Bc).

Finally, we demonstrated that the exogenously expressed full-length MALAT1 could rescue the expression of several of the cell cycle genes in the absence of endogenous human MALAT1 (Figure S2Bd), supporting the involvement of MALAT1 in regulating the expression of these genes in human fibroblasts.

### MALAT1-depleted human diploid fibroblasts fail to progress through the G1 phase

**Figure 3:**
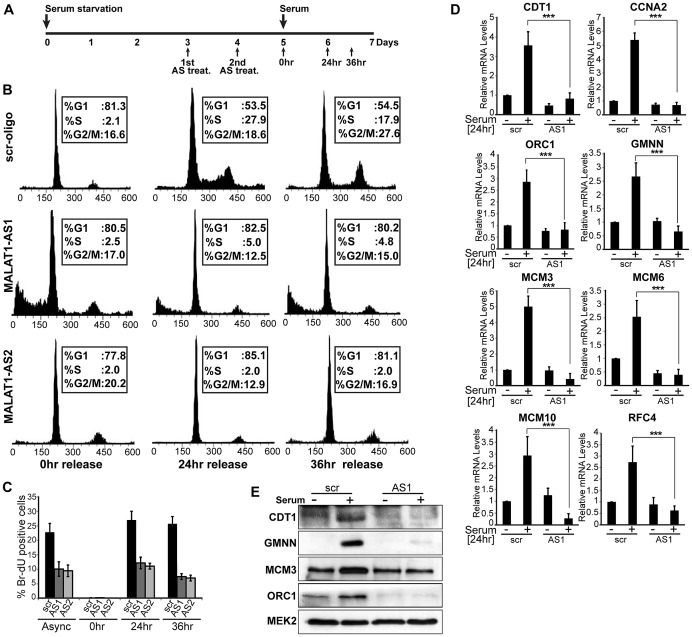
MALAT1-depleted HDFs show defects in G1 to S transition. (A) Flow chart depicting the experimental design. HDFs (WI-38 cells) are G0 arrested by serum starvation, followed by MALAT1depletion (AS treat.) and released from quiescence (G0) by the addition of serum and further examined for G1/S progression in presence or absence of MALAT1. (B–C) Flow cytometry analyses and BrdU-incorporation assays of control (scr-oligo) and MALAT1-depleted cells (AS1 & AS2). (D–E) Effect of MALAT1 knockdown on serum-induced growth control gene expression. Serum-starved WI-38 cells are depleted of MALAT1 followed by serum stimulation, and relative mRNA and protein levels of indicated genes are determined by qPCR and immunoblot analyses. Mean ± SEM,**p<0.01 and ***p<0.001.

MALAT1 is upregulated during the G1/S phase of cell cycle and its depletion showed reduced expression of genes involved in the G1/S transition. This prompted us to examine whether MALAT1 plays a role in G1/S progression. To test this, HDFs were synchronized in G0 (quiescence) by serum starvation and released from quiescence for various time points (0 hr, 24 hr and 36 hr) in the presence or absence of MALAT1 (Figure 3A). qRT-PCR analysis revealed that MALAT1 was significantly depleted at each of the time points analyzed (Figure S3). Flow cytometry data showed that control (scr) oligo-treated cells displayed normal cell cycle progression upon addition of serum (24 and 36 hr after release) (Figure 3B). However, MALAT1-depleted cells (MALAT1-AS1 & -AS2) did not respond to serum and showed defects in S-phase entry with cells accumulated with 2C DNA content (Figure 3B). *In vivo* BrdU incorporation assays also revealed significantly reduced proliferation in MALAT1-depleted cells, indicating cell cycle progression defects (Figure 3C). Finally, in scr-oligo-treated G0 cells, addition of serum induced the expression of genes involved in G1/S transition and S-phase progression, whereas MALAT1-depleted cells failed to activate most of these genes (Figure 3D and 3E). These results suggest that in HDFs, depletion of MALAT1 specifically at G0 prevents the progression of cells into S phase. Our flow cytometry data could not differentiate whether the MALAT1 depleted cells were arrested in G0 or G1 phase of the cell cycle. However, the absence of ORC1, an integral component of the origin recognition complex for DNA replication that is expressed during G1 phase [57], strongly suggests that the cells remained arrested in G0 upon MALAT1 depletion (Figure 3D and 3E).

### p53 is a key downstream mediator of MALAT1

**Figure 4:**
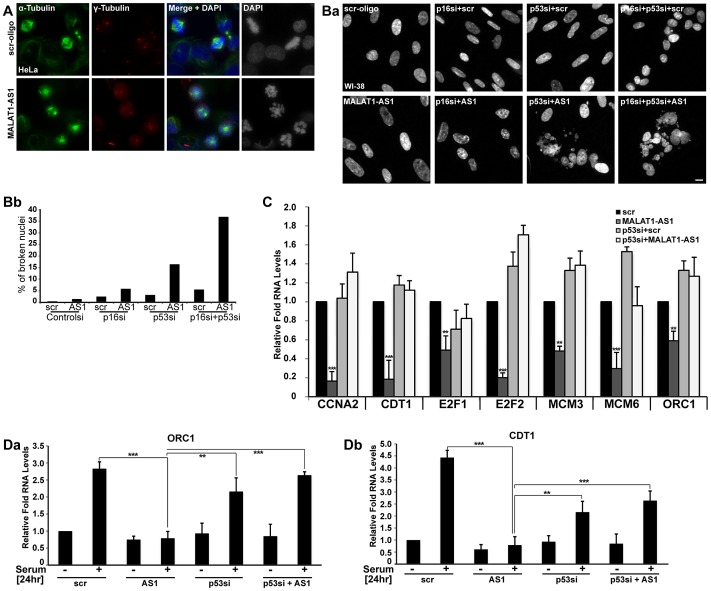
p53 is a downstream mediator of MALAT1. (A) Immuno-localization of α- and γ-tubulin in control (scr-oligo) and MALAT1-depleted (MALAT1-AS1) HeLa cells. Note the presence of monopolar and multipolar asters and highly condensed chromosomes in MALAT1-depleted cells, indicating chromosome segregation defects. (Ba) DNA staining (using Hoechst 33258 dye) in WI-38 cells reveals that co-depletion of MALAT1 and p53 or p53/p16^Ink4A^ results in nuclear breakdown defects. (Bb) Percentage of broken nuclei in control and MALAT1-depleted WI-38 cells in presence or absence of p53 and/or p16^Ink4A^. (C) The relative expression of indicated genes is determined by qRT-PCR analyses from RNA isolated from WI-38 cells that are treated with control or p53 siRNAs and/or control or MALAT1 antisense oligonucleotides. (Da–b) Serum-starved WI-38 cells that are depleted of p53 or MALAT1 or p53 + MALAT1 followed by serum stimulation, and relative mRNA levels of indicated genes are determined by qRT-PCR analyses. The DNA is counterstained with DAPI (A) or Hoechst 33258 (B). Scale bar represents 5 µm. Mean ± SEM,**p<0.01 and ***p<0.001.

MALAT1-depleted HDFs showed a reduction in S-phase cells with a concomitant increase in G1. However, HeLa cells, upon MALAT1 depletion (either using DNA antisense oligonucleotides or siRNAs) showed prominent G2/M arrest with nuclear breakdown phenotype, primarily due to defects in chromosome segregation and spindle assembly (Figure 4A, Figure S4A–S4C). These defects could be partially rescued by the exogenously expressed mouse Malat1, indicating that MALAT1 is involved in mitotic progression (Figure S4Da–b). To determine whether MALAT1 depletion in HeLa cells results in S phase defects (similar to HDFs), we synchronized HeLa cells in mitosis, and released them in presence or absence of MALAT1 and examined the cell cycle progression. We could not arrest HeLa cells in G0 by serum starvation, consistent with the absence of a quiescent state in HeLa cells. Therefore, we synchronized them in prometaphase by nocodazole treatment, transfected with control or MALAT1-specific antisense oligonucleotides and released them for different time points (12, 15 & 18 hrs release) (Figure S4Ea–c). Flow cytometry analyses revealed that both control and MALAT1-depleted HeLa cells showed normal S-phase progression (Figure S4Ea). BrdU incorporation analyses in control and MALAT1-depleted HeLa cells also corroborated the flow data (Figure S4Ec). These results indicate that unlike in normal HDFs, depletion of MALAT1 in HeLa cells did not result in S phase arrest. Since MALAT1-depleted HDFs and HeLa cells showed different phenotypes, we examined the effect of MALAT1 depletion in different cell lines. Indeed, we observed cell line- or cell type-specific responses upon MALAT1 knockdown (Figure S4F). In general, human fibroblasts with a finite life span showed proliferation defects (WI-38, IMR-90 cells), whereas cancer or immortalized cell lines displayed a wide spectrum of abnormalities upon MALAT1 depletion. Surprisingly, MALAT1-depleted HepG2 cells (hepatocarcinoma) did not show any obvious phenotype even though we achieved similar levels of MALAT1 knockdown in these cells. Similarly, depletion of Malat1 in mouse primary (mouse embryonic fibroblasts, MEFs) and transformed fibroblasts (NIH3T3) did not reveal any phenotype (also see [58] (Figure S4F).

Furthermore, detailed analyses revealed that upon MALAT1 depletion, cell lines containing low p53 and/or p16^INK4A^ (*CDKN2A or p16*) activity, including HeLa and U2OS [59], [60] did not display obvious S-phase defects but continued to show severe mitotic abnormalities. We reasoned that depletion of MALAT1 in cells with low p53/p16 activity might not activate the G1/S or intra-S phase checkpoints. In the absence of intact p53, MALAT1-depleted cells, despite having damaged DNA, progressed through G1/S and S phase but eventually arrested in mitosis [50]. To test this possibility, we depleted MALAT1 in WI-38 and WI-38-VA13 subline 2RA cells (WI-38 cells transformed using SV40 T-antigen and as a result have reduced p53 and p16 activity) [61] and examined the cellular phenotype. In contrast to WI-38 cells, MALAT1-depleted WI-38-VA13 cells displayed dramatic mitotic abnormalities and nuclear breakdown phenotype (Figure S4G). Loss-of-function analyses using siRNAs/antisense oligonucleotides against p53, p16 and MALAT1 indicate that WI-38 cells that were co-depleted of MALAT1 along with p53 or p53 and p16 did not show defects in S-phase entry but displayed breakdown of nuclei and mitotic defects (Figure 4Ba–b, Figure S4H). We also examined the alterations in the expression of genes involved in G1/S or S-phase progression in MALAT1 and p53 co-depleted WI-38 cells (Figure 4C). Cells depleted of MALAT1 alone showed reduced expression of several cell cycle genes. However, cells depleted with p53 alone or p53 along with MALAT1 showed normal expression of genes involved in G1/S transition (Figure 4C). We also examined the serum-stimulated response of genes involved in G1/S or S-phase progression in HDFs that were depleted of MALAT1 alone or were co-depleted of MALAT1 along with p53. Unlike the MALAT1 alone-depleted cells, both p53 alone or p53 + MALAT1 co-depleted cells responded to serum by stimulating the expression of genes involved in cell cycle progression (Figure 4Da–b, Figure S4I). These results indicate that functional p53 is vital to evoke G1 or S phase arrest in MALAT1-depleted cells.

**Figure 5:**
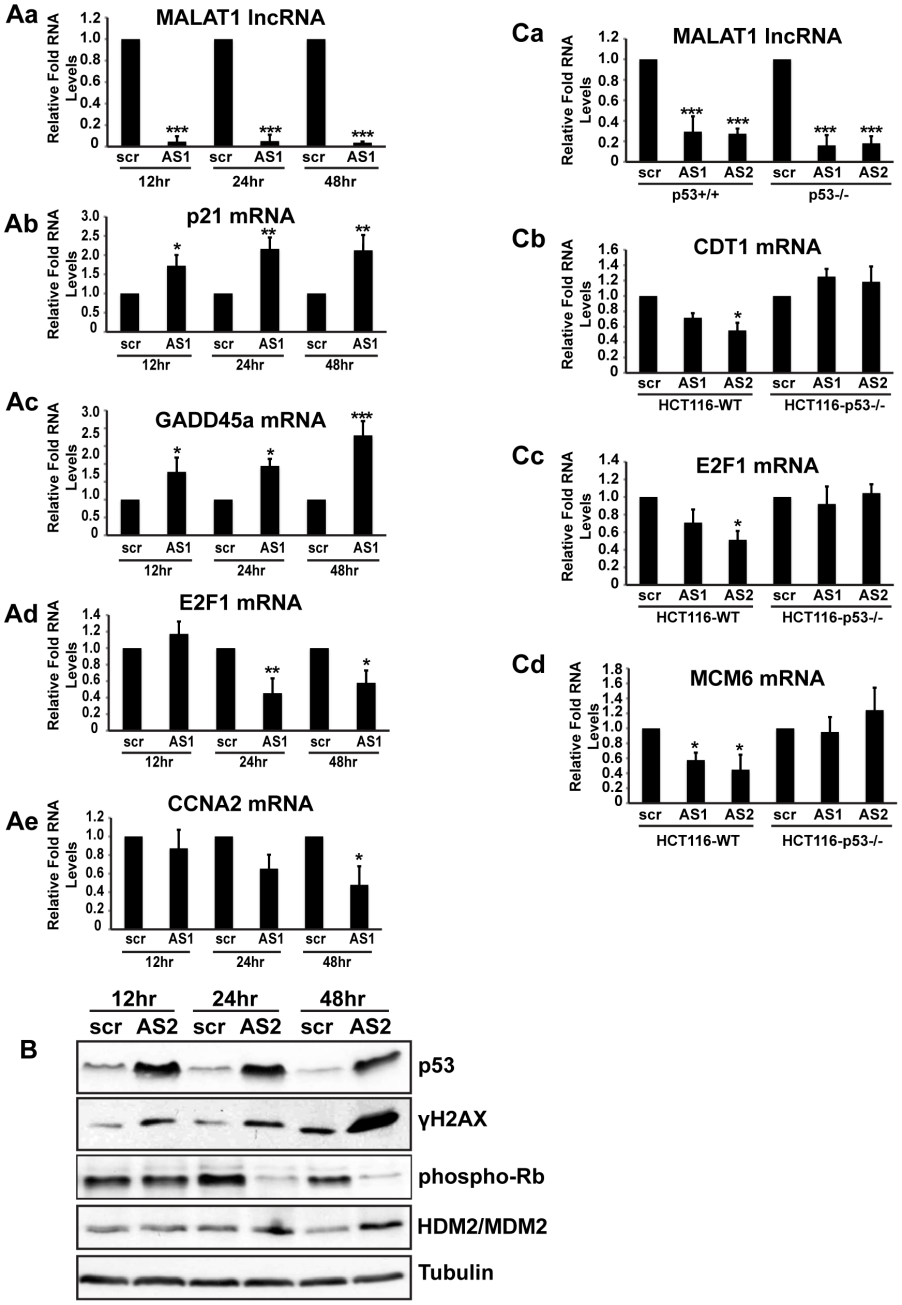
p53 activation is essential for the S-phase defects observed in MALAT1-depleted human cells. (A–B) HDFs (WI-38) are incubated with control (scr) or MALAT1 antisense oligonucleotides (AS1, AS2), and total RNA and protein is isolated at indicated time points (12, 24 & 48 hr). The relative RNA and protein levels of indicated genes are determined by qPCR and immunoblot analyses. Note that the p53 target gene mRNA levels (p21, GADD45a) are increased within 12 hrs of MALAT1 knock down whereas E2F target mRNA (E2F1, CCNA2) levels are reduced only after 24 hrs of MALAT1 depletion. Similarly, MALAT1-depleted cells (all the time points) show increased levels of p53 and γH2AX. However Rb phosphorylation is reduced only 24 hrs after MALAT1 depletion. (C) Relative RNA levels of indicated genes in control (scr) and MALAT1-depleted (AS1 & AS2) HCT116-WT and HCT116-p53 −/− cells. MALAT1-depleted HCT116 p53−/− cells do not show reduction in the levels of E2F target gene mRNAs. Tubulin is used as a loading control. Mean ± SEM, *p<0.05, **p<0.01 and ***p<0.001.

An earlier study reported the involvement of MALAT1 in regulating the activity of E2F1 transcription factor by modulating the PC2 polycomb protein-mediated sumoylation of E2F1 [54]. Both p53 pathway and E2F1-mediated transcriptional activity are interconnected and reduction in E2F1 activity or activation of p53 can elicit similar responses, including cell cycle arrest. To determine which event (reduced activity of E2F1 or activation of p53) is primarily responsible for the cell cycle arrest observed in MALAT1-depleted HDFs, we conducted a time course analysis and assessed p53 and E2F1 activity at different time points after MALAT1 depletion (Figure 5). HDFs showed increased p53 activity within 12 hrs of MALAT1 depletion, as observed by increased p53 levels as well as enhanced expression of p53 response genes, including p21 and GADD45a (Figure 5Aa–c, 5B). Interestingly, cells also showed increased levels of γH2AX within 12 hrs of MALAT1 depletion, suggesting double stranded DNA damage response (DDR) (Figure 5B). These results indicate that induction of p53 upon MALAT1 depletion could be a consequence of double-stranded DNA damage response (DDR). We also examined changes in the expression of E2F1 and its target genes at different time points after MALAT1 depletion. Control and MALAT1-depleted HDFs displayed comparable E2F activity within 12 hrs of MALAT1 knockdown (Figure 5Ad–e, Figure S5Aa–b). However, MALAT1-depleted cells showed reduced levels of E2F1 mRNA and other E2F1 transcription targets after 24 and 48 hr of MALAT1 depletion (Figure 5Ad–e, Figure S5Aa–b). Moreover, control and MALAT1-depleted cells showed similar levels of phosphorylated retinoblastoma (Rb) protein within 12 hrs of MALAT1 silencing. Interestingly, HDFs showed reduced Rb phosphorylation after 24 hr of MALAT1 depletion. Rb is a key factor that controls cell proliferation by regulating progression through the restriction point in G1-phase of the cell cycle. Rb by associating with E2F family proteins negatively regulates E2F transcriptional activity [62]. Cell cycle-dependent hyper-phosphorylation of Rb by cyclin-dependent kinases (CDKs) prevents the association of Rb with E2Fs, allowing cell cycle progression. In MALAT1-depleted cells, p53 accumulation precedes the loss of Rb-phosphorylation. This suggests that the loss of E2F activity upon MALAT1 depletion occurs downstream of p53 activation and is perhaps a consequence of p53-dependent checkpoint activation. How DDR is evoked and how p53 is activated upon MALAT1-depletion remains to be determined. At an earlier time point (12 hr), control and MALAT1-depleted cells showed comparable levels of HDM2/MDM2, a negative regulator of p53 (Figure 5B). However, MALAT1-depleted cells showed increased levels of HDM2 at 24 and 48 hr time points, demonstrating p53-negative feed back loop mechanism.

To determine the importance of p53 activation in the reduced E2F activity and cell cycle arrest observed upon MALAT1 depletion, we compared E2F activity in isogenic wild-type (WT) and p53 −/− HCT116 cells in the presence or absence of MALAT1. Similar to what was observed in HDFs, MALAT1-depleted HCT116-WT cells showed reduced levels of E2F1-target mRNAs (Figure 5Ca–d, Figure S5Ba–c). However, HCT116-p53 −/− cells showed similar levels of E2F-transcribed mRNA in control and MALAT1-depleted cells (Figure 5Ca–d, Figure S5Ba–c). Based on these results, we conclude that a functional p53 is essential for the reduced E2F activity and proliferation defects observed in MALAT1-depleted human cells. It is not clear how MALAT1 influences p53 activity. Transient overexpression of MALAT1 in HDFs did not alter p53 levels (Figure S5Ca–b), indicating that p53 activation in MALAT1-depleted cells could be a part of specific stress response, including DDR.

### Human MALAT1 is required for mitotic progression

**Figure 6:**
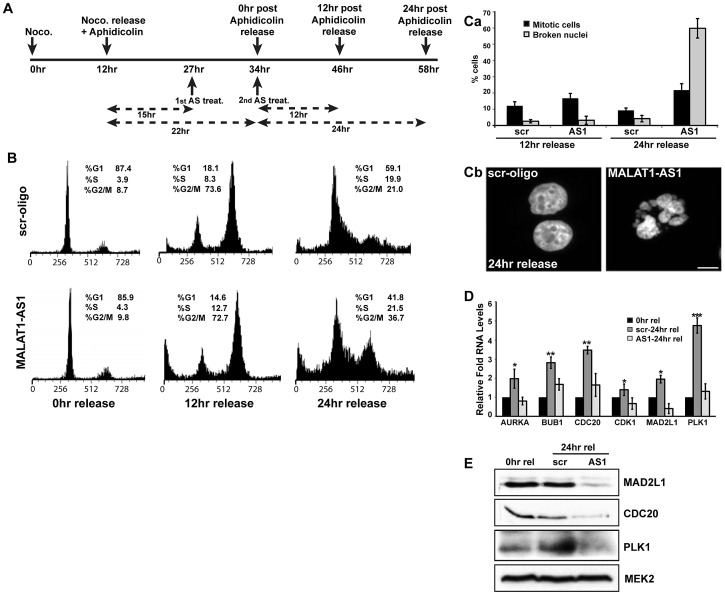
MALAT1 is required for mitotic progression. (A) Flow chart depicting the experimental design. HeLa cells are synchronized in mitosis by nocodazole, released in presence of aphidicolin to synchronize in G1/S. MALAT1 is depleted specifically in G1/S arrested cells and the cells were released for 12 and 24 hr to examine the role of MALAT1 in S-phase and mitotic progression. (B) Flow cytometry analyses of control (scr-oligo) and MALAT1-depleted cells (AS1) post G1/S release. Note the increase in G2/M population in MALAT1-depleted cells (24 hr release). (Ca) Percentage of mitotic cells and cells with broken nuclei in control and MALAT1-depleted cells post G1/S (12 & 24 hr) release. (Cb) Hoechst 33258-stained nuclei in control and MALAT1-depleted HeLa cells post 24 hr G1/S release. (D & E) Effect of MALAT1 knockdown on mitotic gene expression in HDFs. G1/S synchronized WI-38 cells are depleted of MALAT1 followed by release for 24 hr, and relative mRNA and protein levels of indicated genes are determined by qRT-PCR and immunoblot analyses respectively. Note that the MALAT1-depleted cells show reduced expression of mitotic genes compared to scr-oligo-treated cells in post 24 hr release of aphidicolin treatment. MEK2 is used as a loading control. Mean ± SEM, *p<0.05, **p<0.01 and ***p<0.001.

The mitotic defects observed in MALAT1-depleted cells suggested that either MALAT1 has a role in mitosis via regulating the expression/pre-mRNA processing of genes that are involved in mitosis, or the defect observed in p53-deficient cells is a result of damaged DNA or stalled replication forks. Interestingly, mitosis was scored as the most significant canonical pathway of the cell cycle genes that were downregulated in MALAT1-depleted fibroblasts (Figure S6A and S6B). Also, MALAT1 levels were higher during mitosis compared to G2, further supporting the potential involvement of MALAT1 in mitotic progression (Figure 1A). To further examine the potential involvement of MALAT1 in mitotic progression, HeLa (Figure 6A–6C, Figure S6C) or WI-38 (Figure 6D–6E, Figure S6D–S6F) cells were synchronized in G1/S and were released to S-phase in presence of control and MALAT1 antisense oligonucleotides. qRT-PCR results revealed that in G1/S-arrested cells, the levels of MALAT1 were comparable between control (scr) and MALAT1 antisense oligo-treated cells (Figure S6C, scr aphidi. 0 hr rel vs AS1 aphidi. 0 hr rel). However, 12 and 24 hr after G1/S release, cells showed efficient knockdown of MALAT1 (Figure S6C, scr 12 or 24 hr aphidi. rel vs AS1 12 or 24 hr aphidi. rel), indicating that MALAT1 was depleted only in cells beyond G1/S. Flow cytometry analyses showed that the scr-oligo-treated cells showed normal cell cycle progression (Figure 6B). By contrast, while MALAT1-depleted cells showed normal progression in the first 12 hr of release, a significant population of them subsequently accumulated in mitosis by 24 hr after release (Figure 6B, G2/M cells 21% in control vs 37% in MALAT1-depleted cells). Microscopic analyses of the 24 hr-release cells revealed a higher percentage of broken nuclei in MALAT1-depleted cells, indicative of mitotic defects (Figure 6Ca–b). Similar results were also observed in MALAT1-depleted WI-38 cells (Figure S6D–S6F). We also compared the changes in expression of genes involved in mitosis in the 24 hr post G1/S release cells in control (scr-24 hr rel) versus MALAT1-depleted (AS1-24 hr rel) WI-38 cells. In comparison to control cells (scr-24 hr rel), MALAT1-depleted cells (AS1-24 hr rel) showed a consistent reduction in the RNA and protein levels of several of the mitotic genes analyzed (Figure 6D and 6E). Altogether, our results suggest that in human cells, MALAT1 plays a crucial role in mitotic progression that is independent of its involvement in G1 to S transition.

### MALAT1 regulates mitosis by controlling the expression of transcription factor B-MYB (Mybl2)

**Figure 7:**
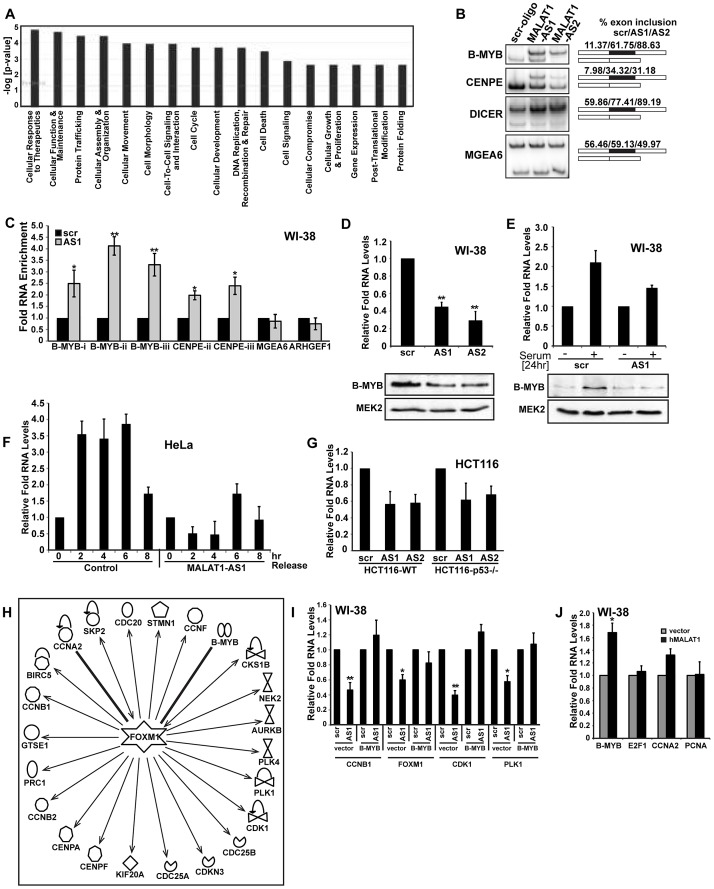
MALAT1 regulates mitotic gene expression by controlling the levels of B-MYB. (A) Top significant biological processes for genes whose alternative splicing was affected in MALAT1-depleted HDFs. (B) RT-PCR analysis using primers specific to exons in MALAT1-regulated alternative exons in indicated mRNAs show changes in alternative splicing in MALAT1-depleted WI-38 cells. Alternative exon-included (upper band) and -excluded bands (lower band) and the percentage change of alternative exon inclusion between scr-oligo/MALAT1-AS oligos (AS1 and AS2) observed is shown. Note that the alternative splicing of MGEA6 pre-mRNA is not altered upon MALAT1 depletion. (C) qRT-PCR analyses of SRSF1 RNA-IP samples from control and MALAT1-depleted (AS1) WI-38 cells reveal increased association of SRSF1 to B-MYB and CENPE mRNA. B-MYB-i-iii and CENPE-ii-iii correspond to separate sets of primers from independent exons of B-MYB and CENPE mRNA. (D) The relative expression and (E) serum-induced activation of B-MYB is determined by qRT-PCR and immunoblot analyses using RNA and protein from control (scr) and MALAT1-depleted (AS1 & AS2) WI-38 cells. (F) G1/S synchronization (0 hr) and release (2–8 hr) in control and MALAT1-depleted HeLa cells reveal reduced levels of B-MYB upon MALAT1 depletion as observed by qRT-PCR analyses. (G) The relative expression of B-MYB is determined by qRT-PCR analyses using RNA from control (scr) and MALAT1-depleted (AS1 & AS2) HCT116-WT and HCT116 p53 −/− cells. (H) Gene network analyses (Ingenuity pathways) indicate that a large number of mitotic specific genes that are downregulated upon MALAT1 depletion are regulated by B-MYB and FOXM1. Note that all the genes mentioned in this figure are downregulated in MALAT1-depleted fibroblasts. (I) Transient expression of B-MYB rescues the expression of mitotic specific genes in MALAT1-depleted WI-38 cells. (J) Relative expression of indicated genes is determined by qRT-PCR analyses in RNA from vector and MALAT1 overexpressed WI-38 cells. Note the increased B-MYB mRNA levels in MALAT1 overexpressed cells. Mean ± SEM, *p<0.05, **p<0.01 and ***p<0.001.

We previously demonstrated that in cancer cells, MALAT1 modulates alternative splicing of pre-mRNAs and regulates the cellular activity of SR splicing factors [50]. In order to determine whether the aberrant expression of cell cycle genes in MALAT1-depleted HDFs were due to alterations in alternative splicing, we analyzed the genome-wide changes in alternative splicing using exon microarrays in control and MALAT1-depleted HDFs (using two independent antisense oligonucleotides against MALAT1). Approximately ∼15% of genes showed changes in alternative splicing (cassette exon inclusion or exclusion) between control and MALAT1-depleted cells (Table S5), which is consistent with our previous findings in HeLa cells [50]. Surprisingly, GO analyses of the mRNAs with altered alternative splicing revealed that cell cycle was not among the top biological processes affected in MALAT1-depleted cells (Figure 7A). Indeed, we observed changes in alternative splicing of a few of the key mitotic regulators, including CENPE (Figure 7B). A large proportion of the cell cycle genes (involved in both G1/S transition and M phase) whose expression was reduced in MALAT1-depleted cells did not show any change in alternative splicing of their pre-mRNAs, implying that the expression of these genes was regulated at the level of transcription or mRNA stability. We reasoned that MALAT1 could influence the expression of key cell cycle regulators (transcription activator or repressor), and that would in turn result in the alteration of transcription of a large number of downstream cell cycle genes. We consistently observed alternative splicing changes for one such transcription regulator B-MYB in MALAT1-depleted cells (Figure 7B). B-MYB is a transcription factor that is required for the transcription of a large number of genes involved in mitotic progression [63], [64], [65]. To determine the involvement of MALAT1 in the SR splicing factor-mediated alternative splicing of B-MYB pre-mRNA, we examined the interaction of SRSF1 with B-MYB exons in the presence or absence of MALAT1. Ribonucleoprotein immunoprecipitation (RNA-IP or RIP) analysis using SRSF1 antibody followed by qRT-PCR revealed the increased association of SRSF1 to B-MYB exons in MALAT1-depleted WI-38 and HeLa cells (Figure 7C and Figure S7A). In addition to B-MYB, SRSF1 displayed increased association with CENPE exons in MALAT1-depleted cells. Both B-MYB and CENPE transcripts displayed aberrant alternative splicing in MALAT1-depleted cells (Figure 7B–7C). We had previously reported changes in alternative splicing of MGEA6 in MALAT1-depleted HeLa cells [50] but not in WI-38 cells (Figure 7B), indicating that MALAT1 could influence alternative splicing in a cell type specific manner. Interestingly, SRSF1 showed increased association to the alternatively spliced exon in MGEA6 only in MALAT1-depleted HeLa cell extracts (Figure S7A) and not in WI-38 cells (Figure 7C), indicating a positive correlation between changes in exon inclusion and increased association of SRSF1 to specific exonic regions upon MALAT1 depletion. Finally, HeLa cells overexpressing SRSF1 showed increased association of SRSF1 to mRNAs, including B-MYB and CENPE (Figure S7B). These results suggest that reduced cellular levels of MALAT1 enhanced the binding affinity of SRSF1 to specific pre-mRNAs, including B-MYB and CENPE pre-mRNA and influenced alternative splicing. We and others have previously reported that MALAT1-depleted cancer cells show increased levels (including the dephosphorylated pool) of SRSF1 [50], [66]. Therefore, the increased association of SRSF1 on B-MYB and CENPE exons observed in MALAT1-depleted cells could be due to the increased total SRSF1 pool. However, unlike HeLa cells, MALAT1-depleted HDFs (WI-38) and mammary epithelial cells (MCF7) did not show any increase in the total or dephosphorylated pool of SRSF1 (Figure S7Ca–c), indicating that other factor/s along with MALAT1 could contribute to changes in SR protein levels observed in MALAT1-depleted HeLa cells. Interestingly, WI-38 cells in which we co-depleted p53 and MALAT1 showed an increased pool of dephosphorylated SRSF1, further indicating the cooperation between MALAT1 and p53 (Figure S7Cd). Taken together, these results suggest that the changes in alternative splicing of pre-mRNAs observed upon MALAT1 depletion is not entirely due to alterations in the total cellular levels of SR proteins but could be due to alterations in the affinity of SR proteins to specific pre-mRNAs.

Recent studies have demonstrated the role of B-MYB in the recruitment of the transcription factor FOXM1 at the promoter of mitotic genes in G2 phase and their transcription as B-MYB-depleted cells fail to recruit FOXM1 to the mitotic gene promoters and showed reduced mitotic gene expression and mitotic defects [64], [67]. Depletion of B-MYB is also known to destabilize FOXM1 transcripts [64]. MALAT1-depleted HDFs showed reduced levels of B-MYB mRNA and protein (Figure 7D and 7E) with a concomitant reduction in FOXM1 transcript levels (Figure S2Ab). B-MYB is transcriptionally induced during G1/S and S-phase of the cell cycle; therefore, reduced levels of B-MYB in MALAT1-depleted fibroblasts could be due to the defects in G1/S and S-phase progression [63], [64]. To determine this, we examined the levels of B-MYB in control and MALAT1-depleted HeLa cells since MALAT1-depletion in HeLa cells did not show defects in S-phase entry (Figure S4E). We observed reduced levels of B-MYB in MALAT1-depleted HeLa cells, indicating that the reduction in B-MYB levels upon MALAT1-depletion is not a reflection of defects in S-phase (Figure S7D). We have also confirmed this by conducting a time course experiment where HeLa cells were synchronized in G1/S, treated with control and MALAT1-specific antisense oligonucleotides and released so that the cells proceed to S and G2 phase. The levels of B-MYB mRNA were subsequently determined at various time points (Figure 7F and Figure S7E). In control cells, B-MYB mRNA level was increased during 2, 4, 6 hr post G1/S-release and showed a reduction at 8 hr post release from G1/S (Figure 7F) [64], [68]. However, MALAT1-depleted post G1/S cells, though showed normal S-phase progression, failed to activate B-MYB and continued to show reduced levels of B-MYB (Figure 7F and Figure S7E). These results reveal that cellular levels of MALAT1 control the expression and/or RNA processing of B-MYB.

B-MYB is an E2F1 target gene. It is possible that the changes in B-MYB expression observed in MALAT1-depleted cells could be due to reduced E2F1 activity. To determine this possibility, we compared the levels of B-MYB mRNA in HCT116 WT and p53 −/− cells in the presence or absence of MALAT1. Both WT and p53 −/− cells showed reduction in B-MYB mRNA levels upon MALAT1 depletion (Figure 7G). This was in contrast to other E2F target genes, whose expression remained unaltered in MALAT1-depleted p53 −/− cells (Figure 5Ca–d, Figure S5Ba–c). Furthermore, exogenously expressed E2F1 could not rescue B-MYB mRNA levels in MALAT1-depleted HDFs, indicating that the reduced B-MYB levels in MALAT1-depleted cells is not due to alterations in E2F1 activity (Figure S7F). Based on these results, we conclude that MALAT1 regulates B-MYB mRNA levels by controlling specific post-transcriptional events.

Pathway analyses indicate that the majority of mitotic genes downregulated in MALAT1-depleted fibroblasts were activated by FOXM1 and that B-MYB regulated this process (Figure 7H). Based on our results, we hypothesize that MALAT1 modulates the FOXM1-mediated transcription of mitotic genes by controlling the expression of B-MYB. To test this model, we conducted a rescue experiment where we transiently expressed B-MYB cDNA in MALAT1-depleted fibroblasts and examined the expression of mitotic genes. Cells transiently expressing B-MYB rescued the expression of FOXM1 and several of its targets (Figure 7I). These data demonstrate that B-MYB acts downstream of MALAT1 and the altered expression of a large number of mitotic genes in MALAT1-depleted fibroblasts are due to reduced levels of B-MYB. To determine whether the cellular levels of MALAT1 truly influence B-MYB expression, we overexpressed MALAT1 in fibroblasts and examined the relative changes in B-MYB mRNA levels. MALAT1-overexpressing cells showed an increase in the levels of B-MYB mRNA (Figure 7J and Figure S7G). At the same time, MALAT1 overexpression did not alter the levels of several other genes that are involved in cell proliferation (including E2F1 and PCNA), indicating that the cellular levels of MALAT1 specifically influence the expression of B-MYB.

## Discussion

The human genome contains a large number of lncRNAs that are dynamically expressed in tissue-, differentiation- and cell type-specific patterns. LncRNAs influence the expression and intracellular distribution of specific proteins and also recruit factors of the chromatin-modifying complexes to specific chromatin sites [4], [9], [10], [13], [15]. Aberrant expression of several lncRNAs is associated with cancer [28], [35], [37], [69]. In the present study, we examined the involvement of the abundant nuclear-retained lncRNA MALAT1 in cell cycle progression. MALAT1 is overexpressed in several cancer types, including lung, breast, colon and hepatocarcinoma, and overexpression of MALAT1 in various cell lines enhanced cell proliferation whereas in nude mice, increased levels of MALAT1 promoted tumor formation [28], [42], [52], [70], [71], [72], [73]. Recent studies have also demonstrated that depletion of MALAT1 impairs proliferative and invasive properties of cancer cells [52], [53], [70], [73]. These results imply that MALAT1 acts as a pro-proliferation gene. However, it was not clear how MALAT1 controls cell proliferation.

In human cells, MALAT1 interacts with several pre-mRNA splicing factors, including SR splicing factors, and MALAT1 influences alternative splicing of pre-mRNAs by regulating the distribution and activity of SR splicing factors. In general, SR splicing factors are constitutively expressed in all cell types and cell cycle stages. Despite this fact, alternative splicing is regulated in a cell type- or cell cycle-specific manner [74], [75]. Based on our recent observations, we propose that differential expression of MALAT1 in different cell types or cell cycle phases, acts as a ‘molecular sponge’ to titrate the cellular pool of SR splicing factors. This in turn creates a gradient of functionally competent splicing factors in the cell ultimately controlling alternative splicing. In support of this model, we demonstrated a cell cycle-regulated expression of MALAT1. Cellular levels of MALAT1 were high during G1/S and M; two important periods of the cell cycle where a cell either commits to enter DNA replication phase or undergoes cell division (M). Depletion of MALAT1 in human diploid cells compromised G1-to-S transition and mitotic progression. Transcriptome microarray in MALAT1-depleted human cells revealed downregulation of genes involved in G1/S and mitotic progression, further supporting the involvement of MALAT1 in cell cycle progression.

A recent study proposed a role for MALAT1 in cell cycle progression through its involvement in regulating E2F1 activity [54]. The authors suggested that MALAT1 modulates the interaction of unmethylated Pc2, a polycomb protein, with E2F1 in serum-activated cells, and such an association facilitates E2F1 SUMOylation, leading to the activation of serum-induced genes [54]. Further, MALAT1-depleted cells showed proliferation defects and an inability to activate E2F target genes upon the addition of serum. Based on these results, the authors proposed that the proliferation defects observed in MALAT1-depleted cells are due to the inactivation of E2F1 [54]. Similar to the previous study, we also observed cell cycle arrest and reduced expression of E2F target genes upon MALAT1 depletion only in specific cell lines (example includes HDFs). In these cells, MALAT1 depletion also resulted in the activation of p53 and its downstream target genes, including p21. It is well established that p53 induction results in cell cycle arrest, cellular senescence or apoptosis [76]. We speculate that the S-phase defects and activated cellular senescence observed in MALAT1-depleted fibroblasts is due to p53 activation and not due to E2F1 inactivation. Our conclusion is based on the observation that HeLa, U2OS, or WI-38-VA13, cells that display weak p53, p16 and Rb activity and retain normal E2F1 function (HeLa due to HPV18 infection, U2OS due to increased HDM2/MDM2 levels and WI-38-VA13 due to SV40 T-antigen) [59], [60], did not show G1 or G1/S arrest upon MALAT1 depletion. Additionally, the G1 arrest in MALAT1-depleted diploid fibroblasts could be successfully rescued by simultaneous depletion of p53. Furthermore, MALAT1 and p53 co-depleted HDFs and MALAT1-depleted HCT116 p53 −/− cells expressed E2F target genes involved in G1/S or S phase progression, indicating normal E2F activity. Finally time course study in MALAT1-depleted HDFs clearly demonstrated that p53 is activated prior to Rb dephosphorylation and E2F inactivation as is evident by the reduced expression of E2F target genes. These results strongly support our model that in normal diploid human lung fibroblasts, the G1- and S-phase defects observed upon MALAT1 depletion is primarily due to p53-mediated checkpoint activation. It remains to be determined how an lncRNA like MALAT1 influences p53 signaling pathway. It is possible that MALAT1 and p53 share a synthetically lethal relationship, where the silencing of one gene (MALAT1) is lethal in the context of the mutation in the second gene (p53). Such a synthetic lethal relationship has been previously reported for cdk2 and MYCN [77]. In this case, depletion of cdk2-induced apoptosis in neuroblastoma cells that overexpressed MYCN, whereas cdk2-depleted neuroblastoma cells with normal MYCN expression did not show any phenotype [77]. It is interesting to note that recent studies have demonstrated the role of other lncRNAs as the potential mediators of p53 signaling network [29], [40], [41].

Our study has demonstrated a role for MALAT1 in mitotic progression that is independent of its involvement in G1/S progression. Chromosome segregation defects were observed in cells even when MALAT1 was depleted in post G1/S stage of the cell cycle, indicating that the mitotic phenotype is not a consequence of defects in G1/S transition. MALAT1-depleted cells showed defects in the expression of a large number of genes involved in mitotic progression. At the same time, only a limited number of mitotic genes, including CENPE and B-MYB, displayed defects in alternative splicing upon MALAT1 depletion. CENPE is a kinesin-like motor protein that is specifically expressed during the G2 phase of cell cycle [78]. It localizes to kinetochores in pro-metaphase cells and plays a crucial role in both chromosome segregation and spindle elongation [78], [79], [80]. Altered splicing and reduced expression of CENPE in MALAT1-depleted cells could contribute to the mitotic defects. On the other hand, B-MYB is a transcription factor with known cell cycle control functions [63]. It is one of the dominant gene signatures for highly proliferative cells observed across multiple tumor types and is highly overexpressed in several cancers [81], [82], [83]. Recent studies demonstrated that during S-phase, B-MYB associated with the multiprotein MuvB complex and localized to the promoters of genes that are expressed during mitosis [64]. Further, the B-MYB-MuvB complex efficiently recruited FOXM1 transcription factor to the mitotic gene promoters and activated their transcription [64]. MALAT1-depleted cells showed a reduction in the cellular levels of B-MYB and its mitotic targets. Finally, exogenously expressed B-MYB rescued the expression of several of the mitotic genes in MALAT1-depleted cells. Based on these results, we suggest that the reduced expression of B-MYB in MALAT1-depleted cells is the major effector leading to aberrant mitotic gene expression. Interestingly, recently published ChiP-seq data reveals that B-MYB binds to the MALAT1 promoter and could potentially regulate MALAT1 expression, indicating that MALAT1 and B-MYB could be part of a positive regulatory loop [64].

In addition to the reduction in B-MYB and CENPE mRNA levels, MALAT1-depleted cells also showed changes in alternative splicing of B-MYB and CENPE transcripts and increased binding of SRSF1 to the exons of these transcripts. The change in alternative splicing of B-MYB and CENPE observed in MALAT1-depleted cells phenocopies that observed in cells overexpressing SRSF1. It is possible that the dynamic changes in MALAT1 levels during the cell cycle titrate the intracellular pool of SR proteins and its association with pre-mRNAs, which in turn influence alternative splicing, stability and expression of specific mRNAs, including B-MYB and CENPE. A recent study revealed differential binding of U2AF65 with MALAT1 in cells when the chromatin structure was perturbed [51]. Global increase in histone hyperacetylation decreased the recruitment of U2AF65 to pre-mRNAs, coincident with an increase in its binding to MALAT1 lncRNA [51]. This further supports the role that MALAT1 acts as a ‘molecular sponge’ to sequester the free pool of splicing factors in the nucleoplasm.

We and others have recently reported that the *in vivo* MALAT1 knockout (KO) mouse is viable and fertile and MEFs from the knock out (KO) mouse did not show any defects in alternative splicing and SR protein activity, indicating that MALAT1 is largely dispensable in mice [58], [84], [85]. The cell type- or organism-specific phenotype observed upon depletion of a particular gene is not specific to MALAT1, as earlier studies had reported similar results for other lncRNAs and protein-coding genes. In human cell lines, the HOTAIR lncRNA transcribed from the HOX C cluster inhibits transcription from HOX D cluster by guiding the recruitment of histone modifiers to specific chromatin in HOX D region [39], [86], [87]. However, a mouse in which the region of HOX C cluster spanning the entire *Hotair* was deleted, showed normal viability and did not show any defects in the HOX D cluster transcription and/or chromatin modifications [88]. Cyclin-dependent kinase 2 (cdk2), a kinase that along with cyclin E is known to play an important role in cell proliferation and G1/S transition, is another classical example of a gene that exerts a cell type- or cell line-specific phenotype upon its depletion. The *in vivo* cdk2 knock out mouse is viable and does not show any obvious defects in normal mitotic cell division cycles [89], [90]. However, depletion of cdk2 showed severe S-phase defects in several human cell lines (HeLa [cervical carcinoma], NCI-H1299 [non-small cell lung carcinoma], A375 & SKMEL5 [both melanoma]) [91], [92], [93], [94] but not in others (SW480 and HT-29 [both colon carcinoma]) [93], [95]. Similar to cdk2, *in vivo* KO mice for several other genes involved in cell proliferation (cyclin E, Cdk4, cyclin D) clearly lacked phenotypes, but showed cell proliferation defects when they were depleted in human cell lines. Finally, cyclin A2 KO mouse is embryonic lethal, as cyclin A2 is required for proliferation in hematopoietic and embryonic stem cells but is not required for cell cycle progression in mouse embryonic fibroblasts, further supporting the cell type-specific role of cell cycle regulators [96]. MALAT1 is involved in a cell type- or tissue type-specific function; accordingly, its depletion shows defects only in specific cells from those tissue lineages. In this context it is known that in mouse, MALAT1 shows a tissue-specific and developmental state-specific expression [43], [58]. Further, the genes whose expression is known to be controlled by MALAT1 could function in a cell type- or tissue type-specific manner. For example, B-MYB knockdown in fibroblasts induces cell cycle arrest and apoptosis [97], [98]. However, B-MYB is found to be dispensable for the proliferation of glioblastoma cells [65].

We have shown that in human cells MALAT1 positively regulates the expression of the oncogenic transcription factor B-MYB. It is interesting to note that the aberrant expression of MALAT1 has also been observed in several cancers [42], [72], [99]; whether such altered expression is a cause or an effect of carcinogenesis needs to be determined. Based on our results, we propose that abnormal expression of MALAT1 in specific cell types or tissues results in aberrant alternative splicing leading to misexpression of genes that are involved in cell cycle progression and/or cell death, thereby contributing to tumor progression.

## Materials and Methods

### Cell culture

HeLa, U2OS, HepG2, WT-MEFs were grown in DMEM containing high glucose, supplemented with penicillin-streptomycin and 10% fetal bovine serum (FBS) (Hyclone, Logan, UT). WI-38, WI-38-VA13 and IMR-90 cells were grown in DMEM containing high glucose + 10% FBS, and 1% non-essential amino acid (NEA). RKO cells were grown in MEM containing high glucose + 10% FBS (Hyclone). HCT116-WT and p53 −/− cells were grown in McCoy's 5A medium + 10%FBS. NIH-3T3 cells were grown in DMEM containing high glucose supplemented with 10% Bovine calf serum (BCS).

### Antisense oligonucleotide and siRNA treatment

Phosphorothioate internucleosidic linkage-modified DNA antisense oligonucleotides were used to deplete human MALAT1. The oligonucleotides were transfected to cells two times (48 hr) within a gap of 24 hr, at a final concentration of 100 nM, using Lipofectamine RNAimax reagent as per the manufacturer's instructions (Invitrogen, USA). Double-stranded siRNAs (Sigma-Genosys, USA) [100] were also used to deplete MALAT1 from cells at a final concentration of 40–50 nM. Depletion of p53 and p16 were performed by smart-pool siRNAs (Dharmacon, Thermoscientific, USA) at 10 nM final concentration.

### Transcription and alternative splicing microarray analysis

For Microarray Analysis, WI-38 cells were reverse transfected in triplicate with either control (scrambled antisense oligo) or two independent MALAT1 antisense oligonucleotides at a final concentration of 100 nM using Lipofectamine RNAiMax (Invitrogen, USA). Total RNA isolated 48 hr post-transfection, was amplified, labeled and hybridized to Illumina arrays (Refseq-8). Raw hybridization intensity data were log-transformed and normalized to yield Z-ratios, which in turn were used to calculate a Z-ratio value for each gene. The Z-ratio was calculated as the difference between the observed gene Z-ratios for the experimental and the control comparisons, divided by the standard deviation associated with the distribution of these differences [101]. Z-ratio absolute values ≥2.5 were chosen as cut-off values, defining increased and decreased expression, respectively. The complete microarray data is available at http://www.ncbi.nlm.nih.gov/geo/query/acc.cgi?acc=GSE44240.

For exon arrays to determine changes in alternative splicing, total RNA (100 ng) was reverse transcribed and amplified using Ambion WT expression kit following the manufacturer's suggested protocol. Sense strand cDNA was fragmented and labeled using Affymetrix WT terminal labeling kit. Duplicate samples from MALAT1 antisense oligonucleotides transfected cells or a control oligonucleotide were hybridized to Affymetrix human Exon ST 1.0 GeneChip in Affymetrix hybridization oven at 45°C, 60 rpm for 16 hr. The arrays were washed and stained on Affymetrix Fluidics Station 450 and scanned on Affymetrix GeneChip scanner 3000 7G. Data were collected using Affymetrix AGCC software. Statistical and clustering analysis was performed with Partek Genomics Suite software using RMA normalization algorithm. Differentially expressed genes and alternatively splicing events were identified with ANOVA analysis. Genes that are up- or downregulated more than 2-fold and with a p<0.001 were considered significant. Significant genes were analyzed for enrichment for pathways using DAVID bioinformatics database (http://david.abcc.ncifcrf.gov/) and Ingenuity Pathway Analysis software. The exon array data is available at http://www.ncbi.nlm.nih.gov/geo/query/acc.cgi?acc=GSE43830.

For the validation of the Microarray data, RNA was reverse transcribed into cDNA using Multiscribe reverse transcriptase. qRT-PCR was performed using primers for genes that showed ≥2.5-fold change in the Microarray data.

### Data analysis and statistics

Relative levels of gene expression were normalized to GAPDH or 7SK RNA. In the RNA-IP experiments, the relative quantities of IP samples were normalized by individual inputs respectively. Results are represented as mean ± SEM of three independent experiments. Comparisons were performed using two-tailed paired Student t-test. *p<0.05, **p<0.01 and ***p<0.0001. In the microarray analyses, relative quantities of gene expression change were normalized by z-ratio between experimental cell group and control cell group. Comparisons were performed using z-test with SAM protocols, plus ANOVA filtering for the sample groups. Cutoff were made for the significant genes with |z ratio|> = 2.5, z-test *p<0.05, ANOVA p-value< = 0.05 , false discovery rate < = 0.30, as well as the average gene expression values in each comparison groups were not negative. Further functional analyses were performed by using Parameterized Gene Set Enrichment (PAGE) algorithm with ALL of the genes on the array. **p<0.01 and ***p<0.0001.

### BrdU-propidium iodide (PI) flow cytometry

For labeling of S-phase cells, BrdU was added in mid-log phase cells at a final concentration of 50 µM and incubated for 1 hr at 37°C. Cells were harvested and washed with PBS + 1% BSA. Cells were further resuspended in chilled 0.9% NaCl at a cell density of 2×10^6^ cells/ml and fixed in equal volume of ethanol and incubated at −20°C for 1 hr. Further, ethanol was removed and the cells were resuspended and incubated in 2N HCl + 0.5% Triton X-100 solution for 30 min at RT followed by washing in 0.1 M Sodium tetraborate solution. Finally, the cells were resuspended in PBS/0.5%Tween-20 + 1% BSA and incubated with anti-BrdU antibody for 1 hr at RT. The cells were washed again with the same buffer and incubated in PBS + PI/Triton X-100 at 37°C for 15 min. Cells were analyzed on a flow cytometer.

Please see Protocol S1 and Table S6 for additional materials and methods.

## Supporting Information

Figure S1 MALAT1 levels are cell cycle regulated and depletion of MALAT1 results in proliferation defects. (A) MALAT1 and Cyclin E levels in 10 and 15 hr post release serum-starved WI-38 cells are analyzed by qRT-PCR. Note that the MALAT1 levels increased during G1/S phase (G1/S is determined by the increased levels of cyclin E). Mean ± SEM, *p<0.05. (B) Expression of SRSF1 is determined by immunoblotting using extracts from cell cycle-synchronized U2OS cells. (C) Flow cytometry analyses of control (scr-oligo) and MALAT1-depleted (MALAT1-AS1 & AS2 antisense oligonucleotides) WI-38 cells. (D) Flow cytometry analyses of control (control-si) and MALAT1-depleted (using MALAT1-si1, -si2 & -si3 double stranded siRNA oligos) WI-38 cells. (E) Bright field low magnification images of control and MALAT1-depleted HDFs show changes in cellular morphology in MALAT1-depleted cells. (F) The relative expression of indicated genes is determined by qRT-PCR analyses from total RNA of control and MALAT1-depleted (using siRNA oligos) WI-38 cells. Mean ± SEM, *p<0.05, **p<0.01 and ***p<0.001.
(PDF)

Figure S2 S2A (Aa–c) The relative expression of indicated genes determined by qRT-PCR from total RNA isolated from control (scr) and MALAT1-depleted (AS1 & AS2) WI-38 cells. (Ad) Changes in relative expression of indicated genes determined by qRT-PCR using total RNA from control (using control siRNA) and MALAT1-depleted (using MALAT1-specific siRNA) WI-38 cells. Note that MALAT1 depletion using double-stranded siRNA oligos also result in reduced expression of cell-cycle genes. (Ae) The relative expression of PCNA is determined by qRT-PCR (with 3 independent primer pairs) using RNA from control (scr) and MALAT1-depleted (AS1 & AS2) WI-38 cells. Note that MALAT1-depleted cells do not show changes in PCNA mRNA levels. Mean ± SEM, **p<0.01 and ***p<0.001. S2B: (Ba) Top significant biofunctions and (Bb) canonical pathways of the protein-coding genes that are upregulated in MALAT1-depleted fibroblasts. Note that the p53-signaling pathway is activated in MALAT1-depleted lung fibroblasts. (Bc) The relative expression of indicated genes is determined by qPCR using RNA from control (scr) and MALAT1-depleted (AS1 & AS2) WI-38 cells. Note that several of the upregulated genes are part of the p53-signaling pathway (*Cdkn1a [p21]*, *Gadd45a*, *Gadd45b*, *Il-6*, *Il-8*, *Mdm2*, *Tp53inp1*). (Bd) qRT-PCR analyses reveal that transiently expressed MALAT1 rescues the expression of cell cycle genes (*Mybl2*, *Ccna2*, *CenpE*) in MALAT1-depleted HDFs. Mean ± SEM, *p<0.05 and **p<0.01.
(PDF)

Figure S3 The relative levels of MALAT1 are determined by qRT-PCR analyses in RNA from serum starved (0 hr) and serum re-stimulated (24 & 36 hr) control (scr) and MALAT1-depleted (AS1 & AS2) WI-38 cells. ‘Async’ designates asynchronous WI-38 cells.
(PDF)

Figure S4 p53 is a downstream mediator of MALAT1. (A) Co-immunolocalization of α- and γ-tubulin, (B) nucleoporin and lamin A/C in control and MALAT1-depleted HeLa cells. Note the presence of monopolar and highly condensed chromosomes in MALAT1-depleted cells (A). (C) MALAT1 RNA-FISH in control (control-si) and MALAT1-depleted (using MALAT1 siRNAs; si1 & si2) HeLa cells. (Da) MALAT1 RNA-FISH in human MALAT1-depleted HeLa cells that express mouse MALAT1. (Db) Percentage of mouse Malat1 expressing HeLa cells (as observed by MALAT1 RNA-FISH) with normal and broken nuclei upon human MALAT1 depletion. Note that the HeLa cells that express mouse MALAT1 do not show nuclear break down upon depletion of endogenous human MALAT1. (Ea–c) HeLa cells are synchronized in mitosis by nocodazole, incubated with control or MALAT1 antisense oligonucleotides and released for indicated time points to examine the role of MALAT1 in S-phase progression. (Ea) Flow cytometry analyses of control (scr-oligo) and MALAT1-depleted cells (AS1) post-mitotic release. (Eb) The relative MALAT1 RNA levels in control and MALAT1-depleted HeLa cells that are released (12, 15 & 18 hr) post nocodazole treatment. (Ec) BrdU-incorporation assays of control (scr) and MALAT1-depleted cells (AS1) post 12 hr nocodazole release. (F) Table showing the phenotypes (mitosis and cell cycle arrest) observed in several cell lines upon MALAT1 depletion. +++ maximum, ++ medium and + minor changes. ‘M’ designates for mitotic defects and ‘NP’ stands for no obvious phenotype. RKO cells showed cell death upon MALAT1 depletion. (Ga–b) MALAT1 RNA-FISH in control (scr-oligo) and MALAT1-depleted (AS1 & AS2) WI-38 and WI-38-VA13 (WI-38 cells stably expressing SV40-T-antigen). (Ha–c) Relative levels p16^Ink4A^ (Ha), p53 (Hb) and MALAT1 (Hc) RNA is determined by qRT-PCR in WI-38 cells that are transfected with control (scr), p16^Ink4A^ siRNA (p16si), p53 siRNA (p53si), MALAT1 antisense oligos (AS1), or combination of siRNAs with MALAT1 AS1. (I) Q-PCR analyses to determine the relative mRNA levels of MCM6 in serum starved WI-38 cells that are depleted of p53 or MALAT1 alone or p53 + MALAT1 followed by serum re-stimulation. Mean ± SEM, *p<0.05, **p<0.01 and ***p<0.001. The DNA is counterstained with DAPI. The scale bar represents 5 µm.
(PDF)

Figure S5 Functional p53 is required for the S-phase arrest observed in MALAT1-depleted human cells. (A) The relative RNA levels of indicated genes in HDFs that are incubated with control (scr) or MALAT1 antisense oligonucleotides (AS1), and total RNA is isolated at indicated time points (12, 24 & 48 hr). Note that the E2F target mRNA (CDT1, MCM3) level is reduced after 24 hrs of MALAT1 depletion. (B) Relative RNA levels of indicated genes in control (scr) and MALAT1-depleted (AS1 & AS2) HCT116-WT and HCT116-p53 −/− cells. MALAT1-depleted HCT116 p53−/− cells do not show reduction in the levels of E2F target gene mRNAs. (Ca) Relative MALAT1 RNA levels in HDFs that are transiently transfected with control (vector) and human MALAT1 plasmids. (Cb) Immunoblot assay to detect the p53 in vector and human MALAT1 plasmid transfected HDFs. B″-U2snRNP is used as a loading control.
(PDF)

Figure S6 MALAT1 is required for mitotic progression (A) Top significant canonical pathways of the cell cycle genes that are downregulated in MALAT1-depleted fibroblasts. Note that the expression of genes involved in mitotic progression is maximally affected upon MALAT1 depletion. (B) The yellow shaded genes in the pathway analysis diagram are downregulated in MALAT1-depleted fibroblasts, further emphasizing the involvement of MALAT1 in mitotic progression. (C) The relative MALAT1 RNA levels in mitotic (Noco), G1 (Noco 6 hr rel), G1/S synchronized HeLa cells (0 hr rel) and cells that are 12 and 24 hr released in presence of scrambled and MALAT1 antisense oligonucleotides. (D) Flow chart depicting the experimental design. HDFs (WI-38cells) are synchronized in G1/S by serum starvation followed by aphidicolin treatment, followed by MALAT1 depletion and released for 12 and 24 hr to examine the role of MALAT1 in S-phase and mitotic progression. (Ea) Percentage of mitotic cells and cells with broken nuclei in control and MALAT1-depleted HDFs post G1/S (12 and 24 hr) release. (Eb) DAPI-stained nuclei in control and MALAT1-depleted HDFs post 24 hr G1/S release. (F) Immuno-localization of α-tubulin and γ-tubulin in control and MALAT1-depleted WI-38 cells post G1/S (24 hr) release. Note that MALAT1-depleted diploid cells show mitotic segregation defects with monopolar asters. DNA is counterstained with DAPI. Scale bar represents 5 µm.
(PDF)

Figure S7 (A) qRT-PCR analyses of SRSF1 RNA-IP samples from control and MALAT1-depleted HeLa cells reveal increased association of SRSF1 to indicated pre-mRNAs. (B) qRT-PCR analyses of T7 RNA-IP samples from control and T7-tagged SRSF1 over expressed HeLa cells reveal increased association of SRSF1 to indicated pre-mRNAs in T7-SRSF1 overexpressed cells. Mean ± SEM, *p<0.05, **p<0.01 and ***p<0.001. (Ca) Immunoblot analysis of SRSF1 in control (control siRNA oligo = luciferase gene siRNA; scr oligo = scrambled oligo against MALAT1 AS2 oligo), MALAT1-AS2) and Neat1-AS1 antisense oligo-treated HeLa cells. The cells are transfected with various concentrations (50, 100 & 200 nM) of siRNA or modified DNA antisense oligos using Lipofectamine RNAimax transfection reagent. The arrow designates the dephosphorylated SRSF1. (Cb) Immunoblot analysis of SRSF1 in control (control si), MALAT1-depleted (using double-stranded siRNAs; si1 & si2) HeLa cell extracts. (Cc) Immunoblot analysis of SRSF1 in control (scr-oligo) and MALAT1-depleted (AS2), MCF7, HeLa and WI-38 cell extracts. Note that the dephosphorylated SRSF1 is present only in MALAT1-depleted HeLa cells. (Cd) Immunoblot analysis of SRSF1 in control (scr-oligo or control siRNA), p53 or MALAT1 or p53 + MALAT1 co-depleted WI-38 cell extracts. Note that p53 and MALAT1 co-depleted WI-38 cells show increased levels of dephosphorylated SRSF1. (D) qRT-PCR using RNA from control and MALAT1-depleted HeLa cells to determine the relative expression of B-MYB. (E) Flow cytometry analyses of control and MALAT1-depleted G1/S synchronized (0 hr) and released (2–8 hr) HeLa cells reveal comparable cell cycle progression. (Fa–b) The relative levels of indicated mRNAs (E2F1 and B-MYB) in control (scr) and MALAT1-depleted (AS1) HDFs that are transiently transfected with vector or E2F1 eukaryotic expression plasmids. (G) The relative levels of MALAT1 are determined by qRT-PCR analyses in RNA from control and MALAT1 overexpressed WI-38 cells.
(PDF)

Protocol S1 Supporting materials and methods.
(DOCX)

Table S1 Gene expression changes upon knockdown of MALAT1 with Antisense oligo 1 (AS1) in WI-38 cells.
(XLSX)

Table S2 Gene expression changes upon knockdown of MALAT1 with Antisense oligo 2 (AS2) in WI-38 cells.
(XLSX)

Table S3 Common genes downregulated (Z-ratio ≥2.50) upon knockdown of MALAT1 with both antisense oligo 1 (AS1) or antisense oligo 2 (AS2) in WI-38 cells.
(XLSX)

Table S4 Common genes up-regulated (Z-ratio≤2.50) upon knockdown of MALAT1 with both antisense oligo 1 (AS1) and antisense oligo 2 (AS2) in WI-38 cells.
(XLSX)

Table S5 Effect of MALAT1 knockdown on alternative splicing. Antisense oligo 1 (AS1) and Antisense oligo 2 (AS2) are two independent antisense oligonucleotides that were used for MALAT1 knockdown in WI-38 cells.
(XLSX)

Table S6 List of qRT-PCR primers and siRNAs or modified DNA antisense oligonucleotides used in the present study.
(XLS)
