# Supplementary figures and images for: Long Noncoding RNA MALAT1 Controls Cell Cycle Progression by Regulating the Expression of Oncogenic Transcription Factor B-MYB
Source: PLoS Genet. 2013 Mar 21;9(3):e1003368. doi: 10.1371/journal.pgen.1003368 (PMC3605280; doi:10.1371/journal.pgen.1003368)

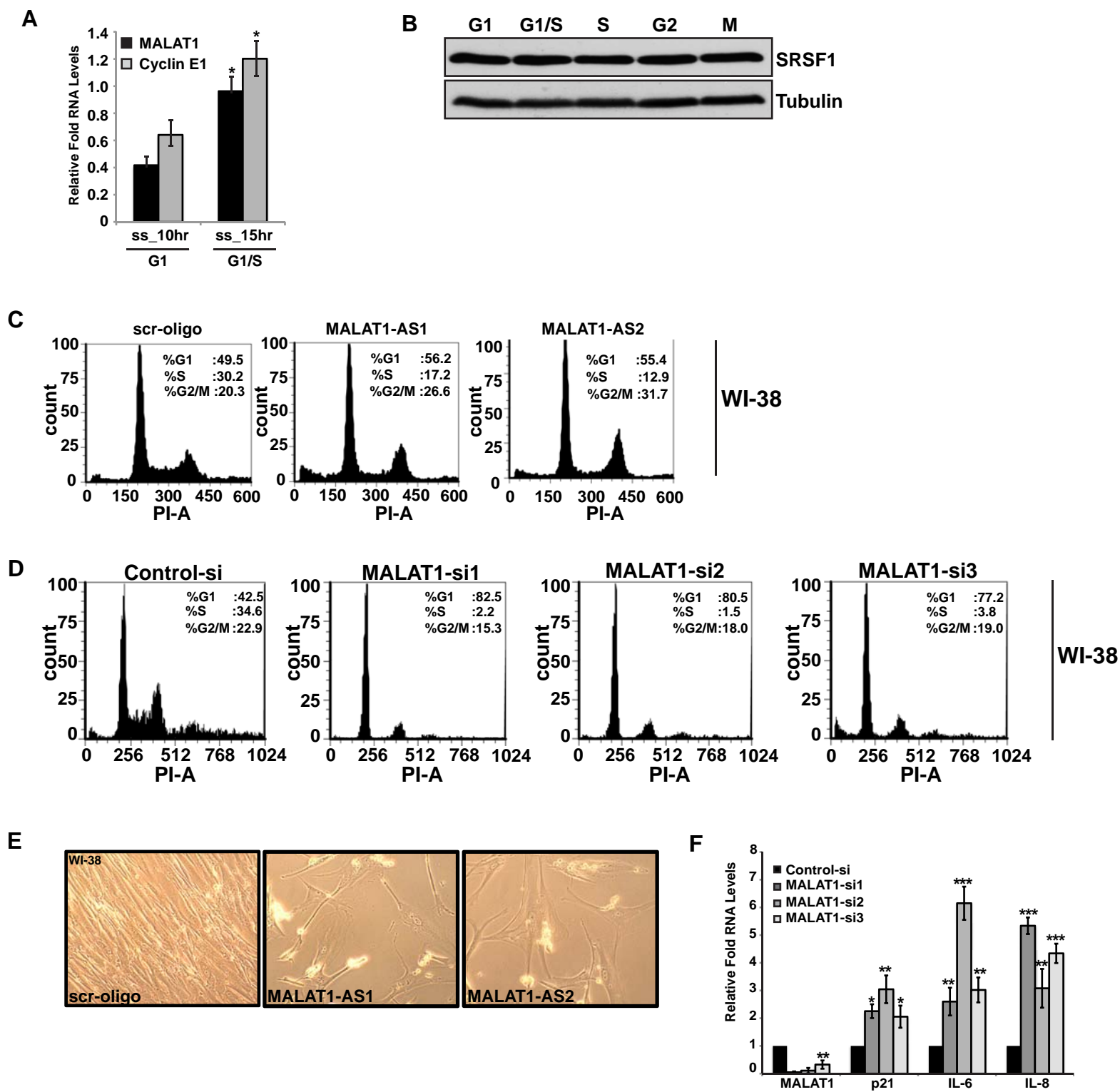

Supplement: Figure S1 — MALAT1 levels are cell cycle regulated and depletion of MALAT1 results in proliferation defects. (A) MALAT1 and Cyclin E levels in 10 and 15 hr post release serum-starved WI-38 cells are analyzed by qRT-PCR. Note that the MALAT1 levels increased during G1/S phase (G1/S is determined by the increased levels of cyclin E). Mean ± SEM, *p<0.05. (B) Expression of SRSF1 is determined by immunoblotting using extracts from cell cycle-synchronized U2OS cells. (C) Flow cytometry analyses of control (scr-oligo) and MALAT1-depleted (MALAT1-AS1 & AS2 antisense oligonucleotides) WI-38 cells. (D) Flow cytometry analyses of control (control-si) and MALAT1-depleted (using MALAT1-si1, -si2 & -si3 double stranded siRNA oligos) WI-38 cells. (E) Bright field low magnification images of control and MALAT1-depleted HDFs show changes in cellular morphology in MALAT1-depleted cells. (F) The relative expression of indicated genes is determined by qRT-PCR analyses from total RNA of control and MALAT1-depleted (using siRNA oligos) WI-38 cells. Mean ± SEM, *p<0.05, **p<0.01 and ***p<0.001. (PDF) [file pgen.1003368.s001.pdf]

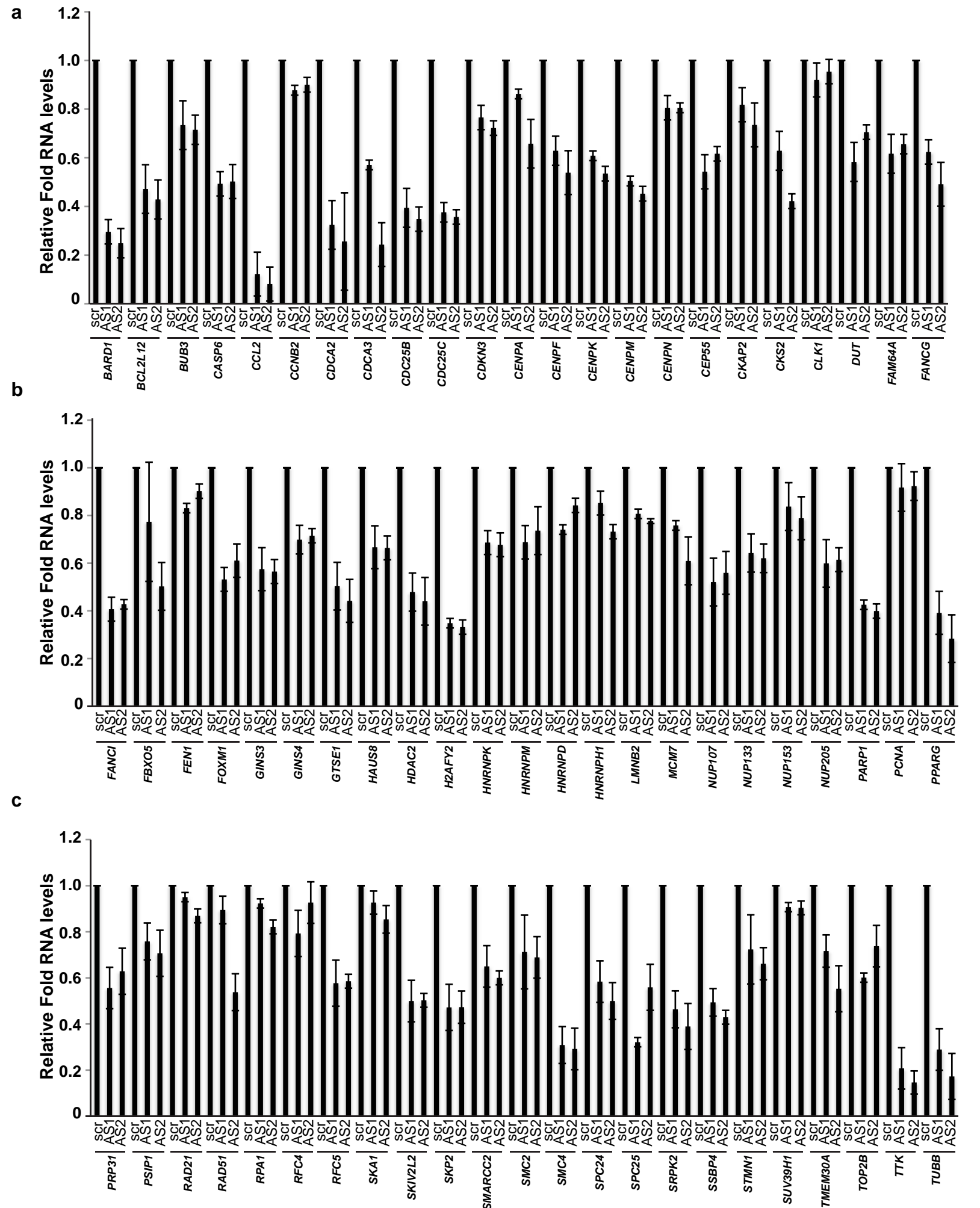

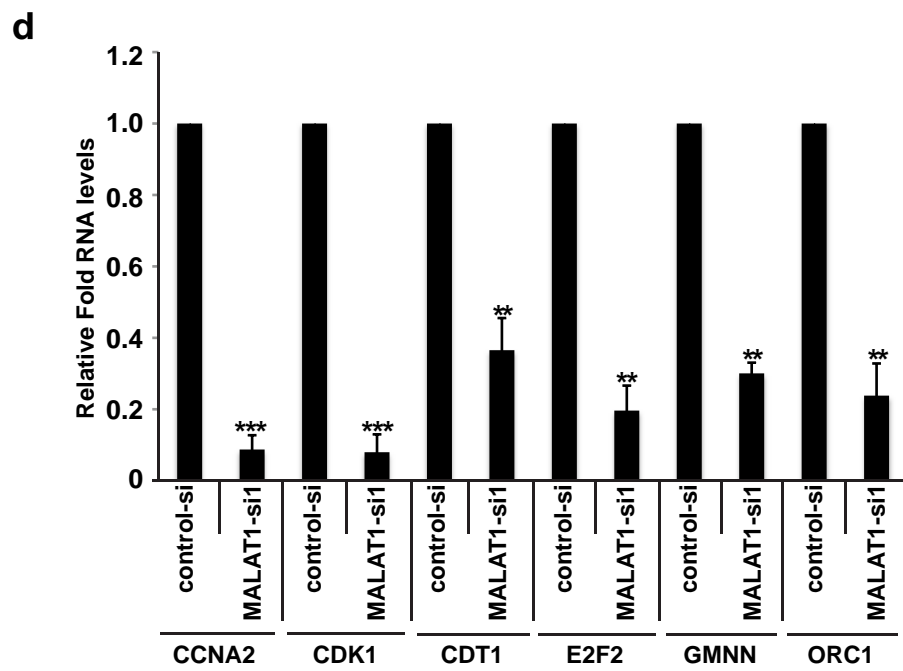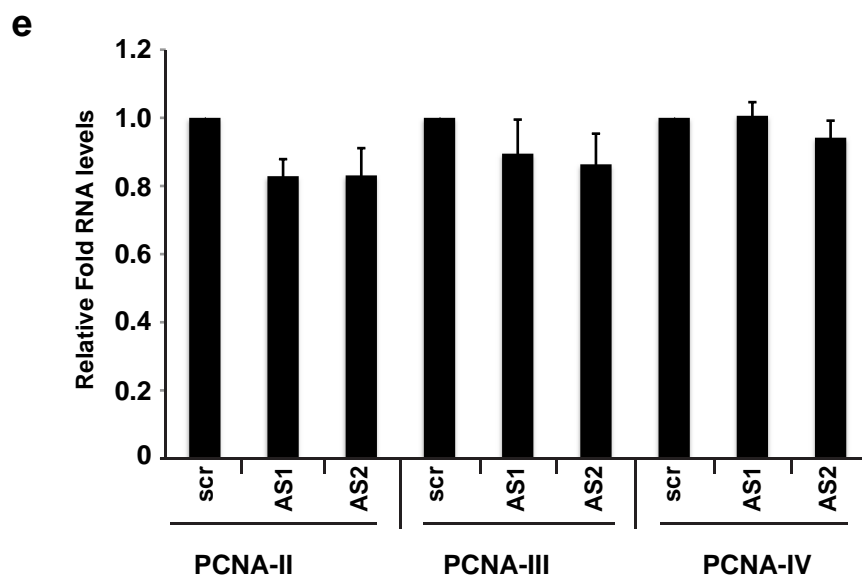

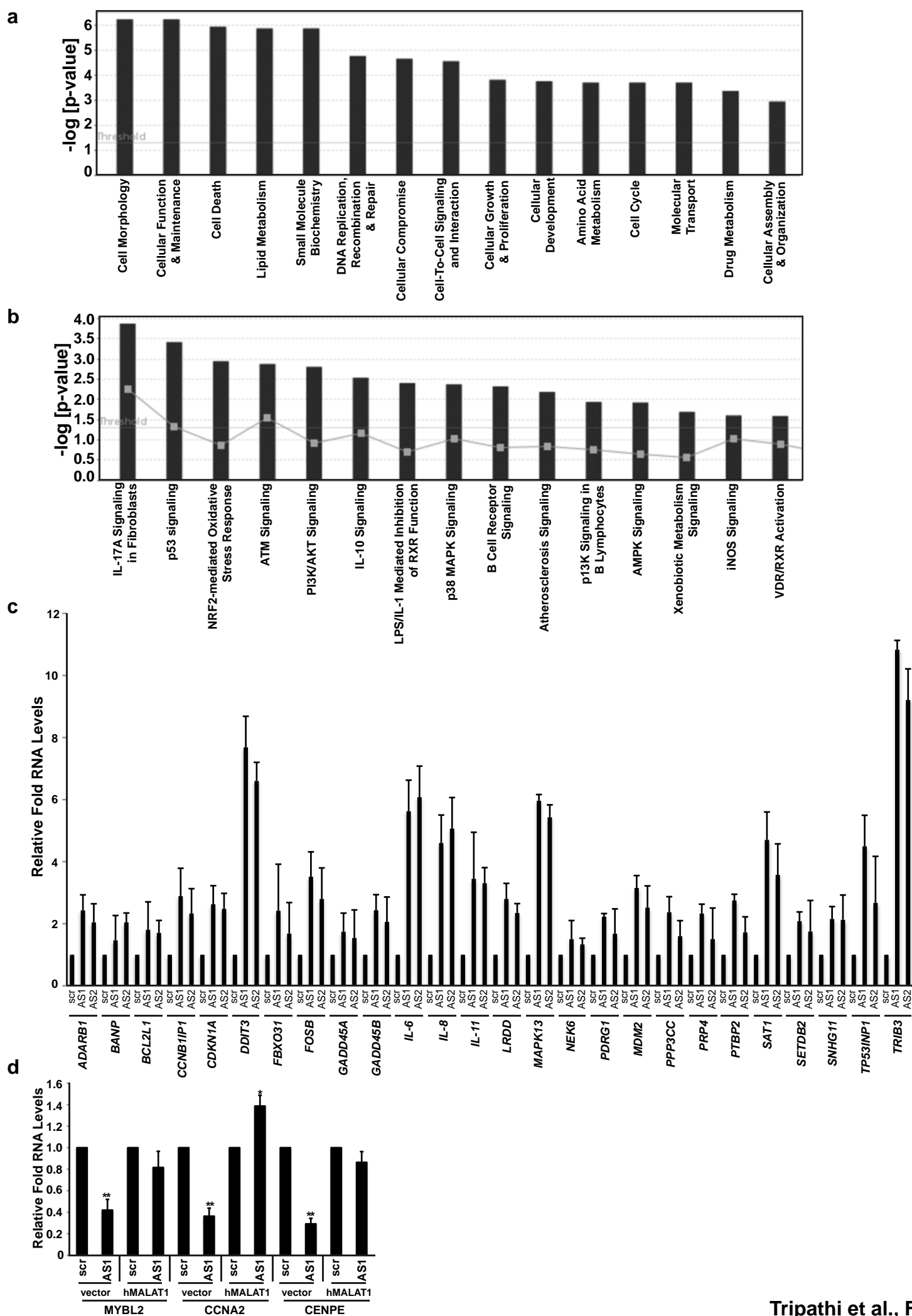

Supplement: Figure S2 — S2A (Aa–c) The relative expression of indicated genes determined by qRT-PCR from total RNA isolated from control (scr) and MALAT1-depleted (AS1 & AS2) WI-38 cells. (Ad) Changes in relative expression of indicated genes determined by qRT-PCR using total RNA from control (using control siRNA) and MALAT1-depleted (using MALAT1-specific siRNA) WI-38 cells. Note that MALAT1 depletion using double-stranded siRNA oligos also result in reduced expression of cell-cycle genes. (Ae) The relative expression of PCNA is determined by qRT-PCR (with 3 independent primer pairs) using RNA from control (scr) and MALAT1-depleted (AS1 & AS2) WI-38 cells. Note that MALAT1-depleted cells do not show changes in PCNA mRNA levels. Mean ± SEM, **p<0.01 and ***p<0.001. S2B: (Ba) Top significant biofunctions and (Bb) canonical pathways of the protein-coding genes that are upregulated in MALAT1-depleted fibroblasts. Note that the p53-signaling pathway is activated in MALAT1-depleted lung fibroblasts. (Bc) The relative expression of indicated genes is determined by qPCR using RNA from control (scr) and MALAT1-depleted (AS1 & AS2) WI-38 cells. Note that several of the upregulated genes are part of the p53-signaling pathway (Cdkn1a [p21], Gadd45a, Gadd45b, Il-6, Il-8, Mdm2, Tp53inp1). (Bd) qRT-PCR analyses reveal that transiently expressed MALAT1 rescues the expression of cell cycle genes (Mybl2, Ccna2, CenpE) in MALAT1-depleted HDFs. Mean ± SEM, *p<0.05 and **p<0.01. (PDF) [file pgen.1003368.s002.pdf]

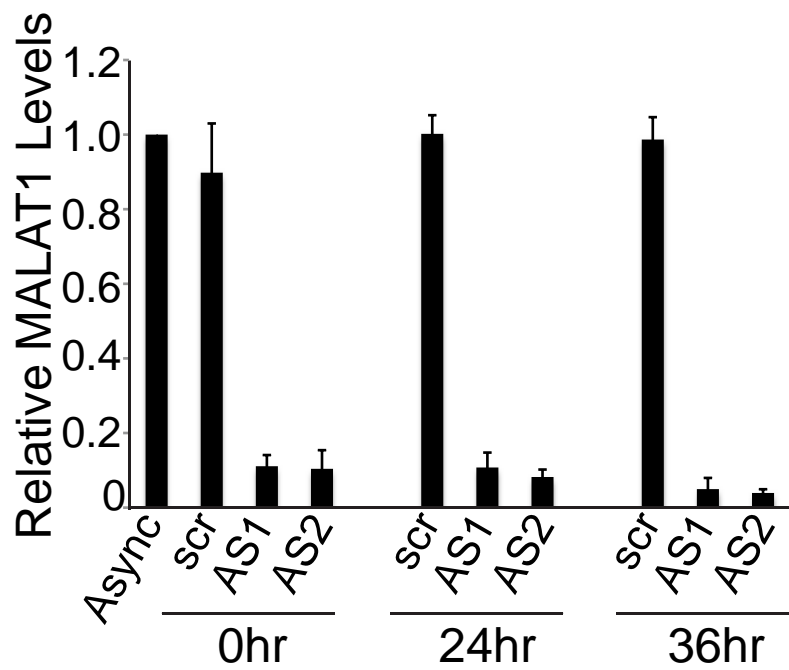

Supplement: Figure S3 — The relative levels of MALAT1 are determined by qRT-PCR analyses in RNA from serum starved (0 hr) and serum re-stimulated (24 & 36 hr) control (scr) and MALAT1-depleted (AS1 & AS2) WI-38 cells. ‘Async’ designates asynchronous WI-38 cells. (PDF) [file pgen.1003368.s003.pdf]

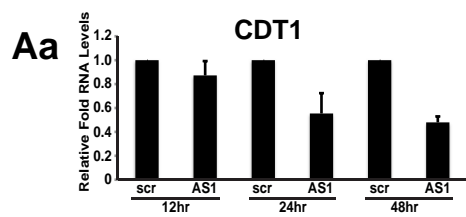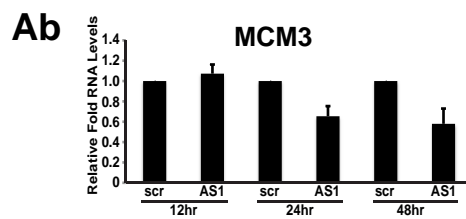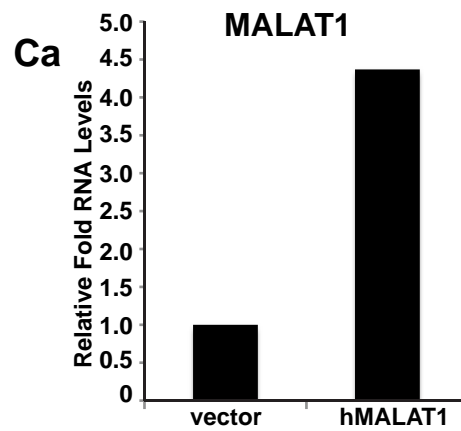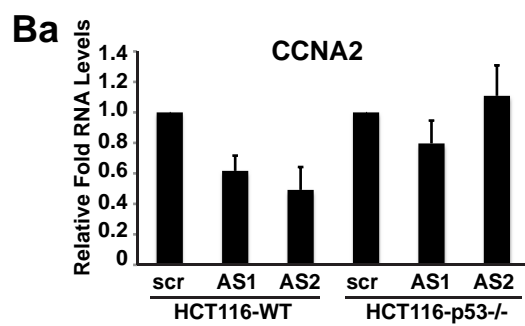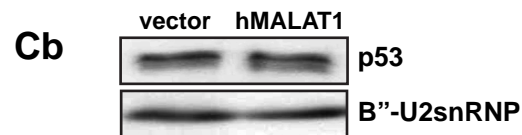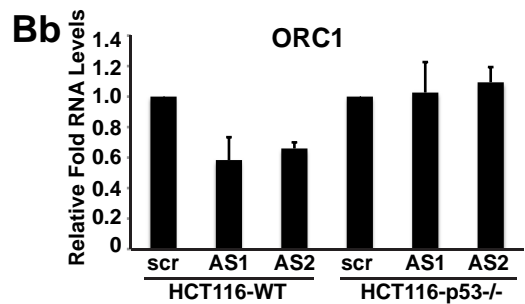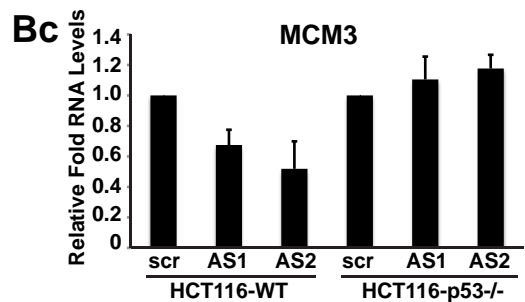

Supplement: Figure S5 — Functional p53 is required for the S-phase arrest observed in MALAT1-depleted human cells. (A) The relative RNA levels of indicated genes in HDFs that are incubated with control (scr) or MALAT1 antisense oligonucleotides (AS1), and total RNA is isolated at indicated time points (12, 24 & 48 hr). Note that the E2F target mRNA (CDT1, MCM3) level is reduced after 24 hrs of MALAT1 depletion. (B) Relative RNA levels of indicated genes in control (scr) and MALAT1-depleted (AS1 & AS2) HCT116-WT and HCT116-p53 −/− cells. MALAT1-depleted HCT116 p53−/− cells do not show reduction in the levels of E2F target gene mRNAs. (Ca) Relative MALAT1 RNA levels in HDFs that are transiently transfected with control (vector) and human MALAT1 plasmids. (Cb) Immunoblot assay to detect the p53 in vector and human MALAT1 plasmid transfected HDFs. B″-U2snRNP is used as a loading control. (PDF) [file pgen.1003368.s005.pdf]

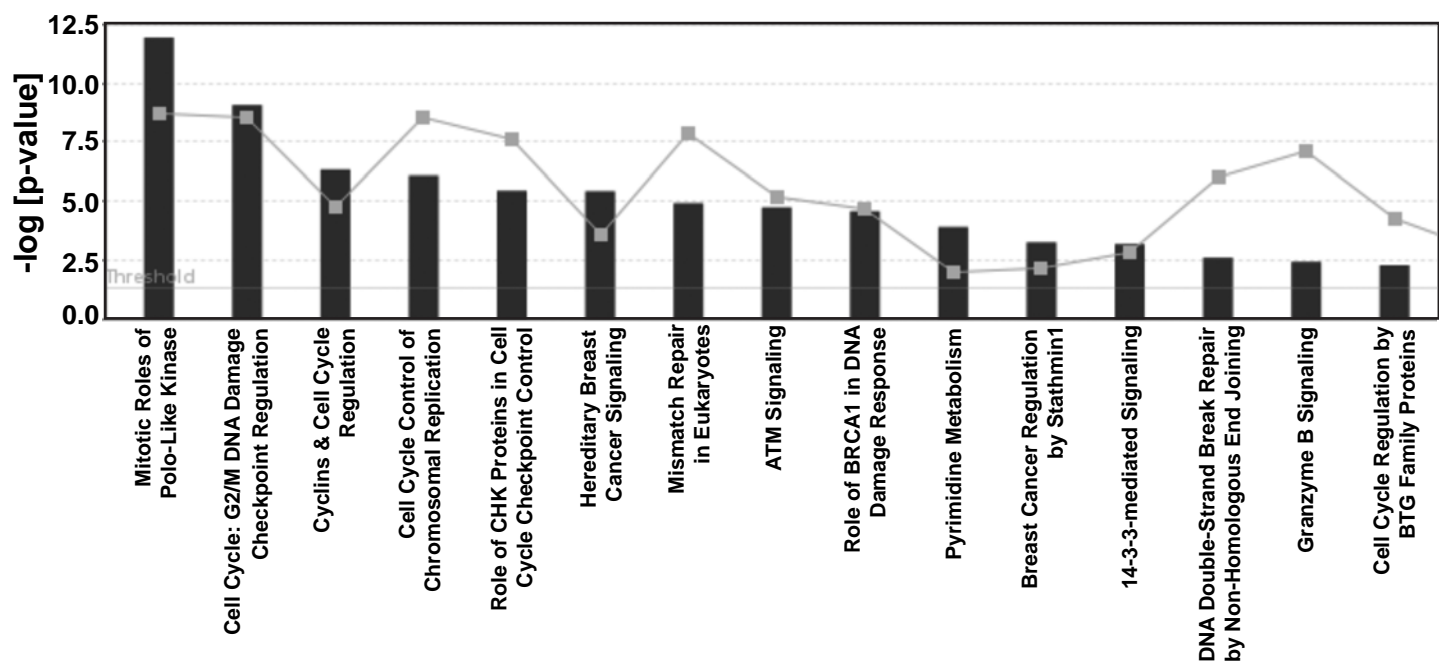

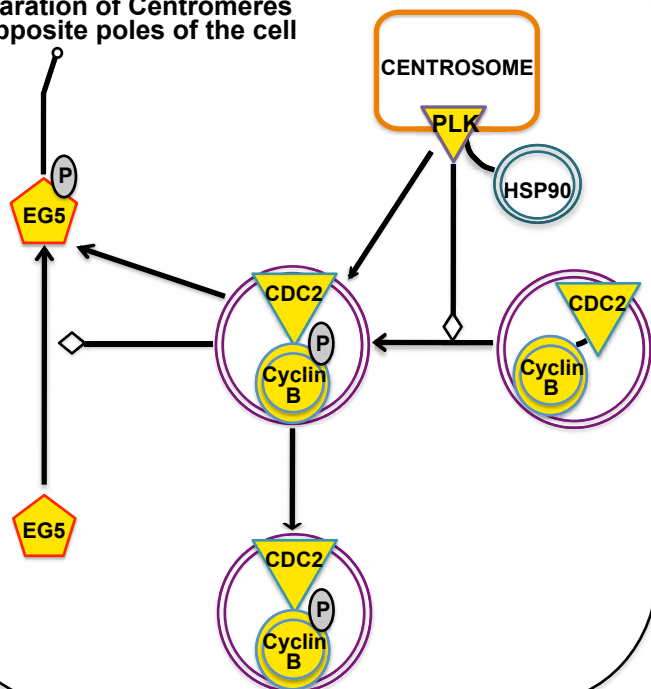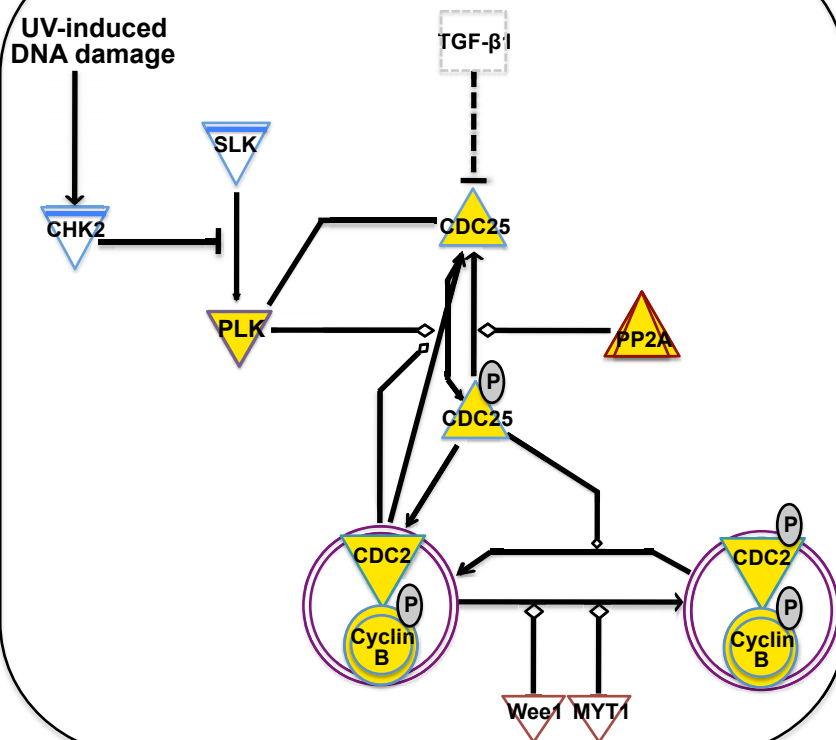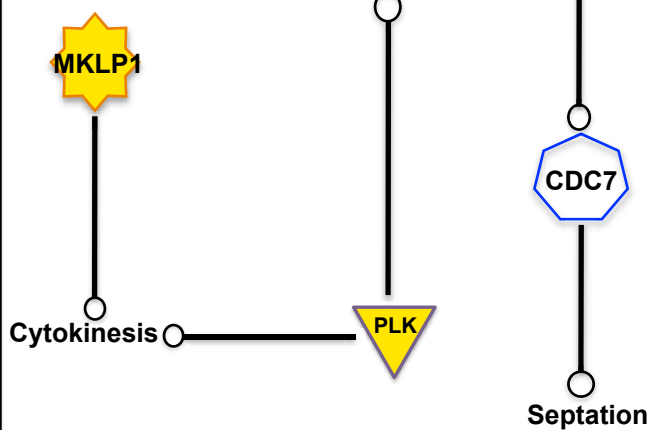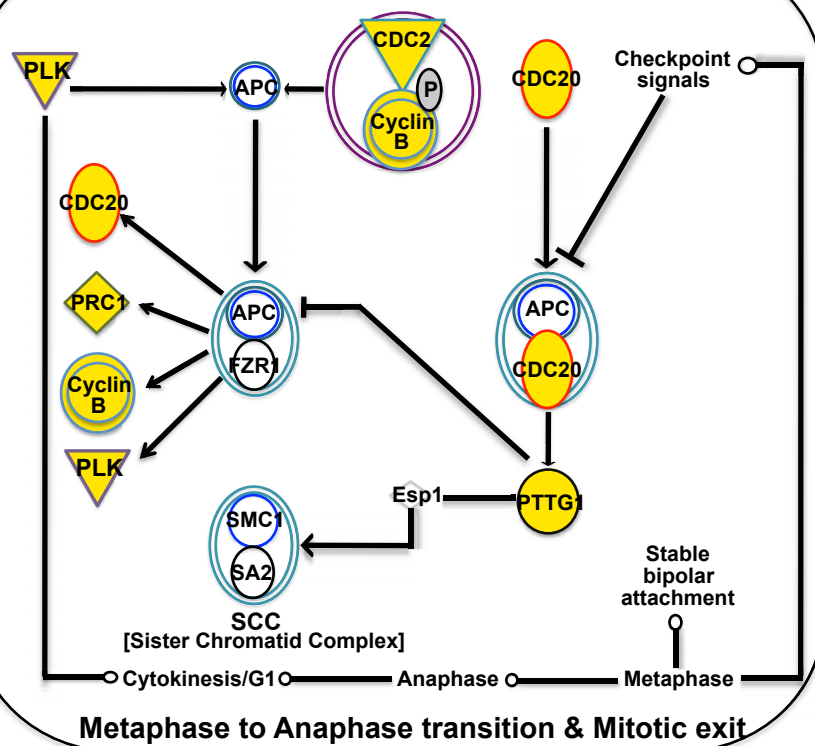

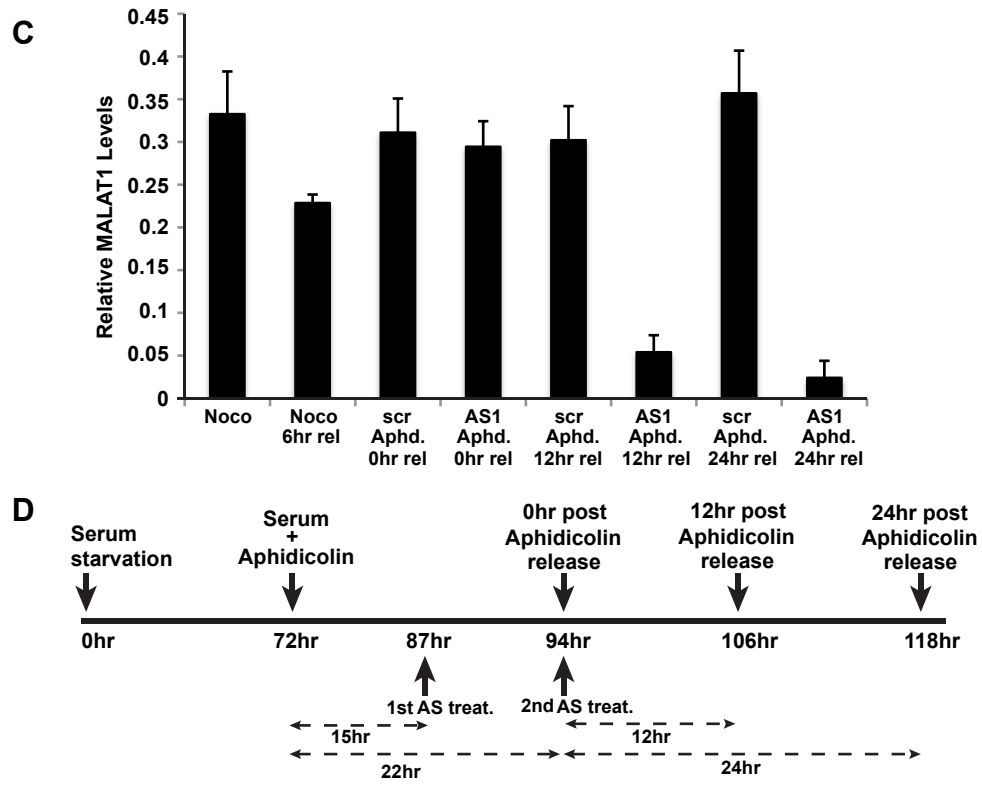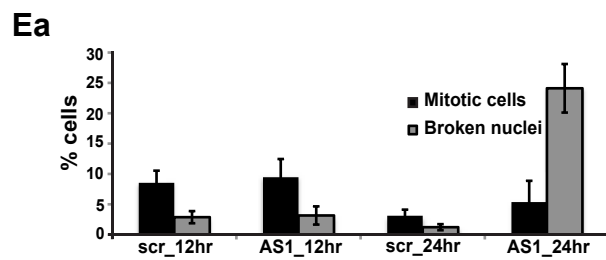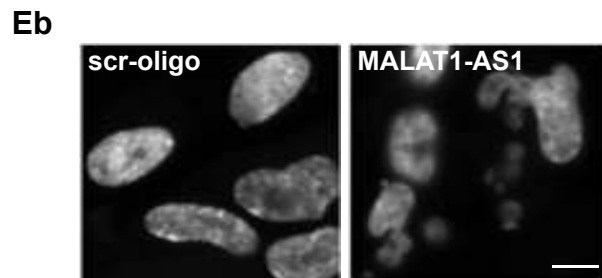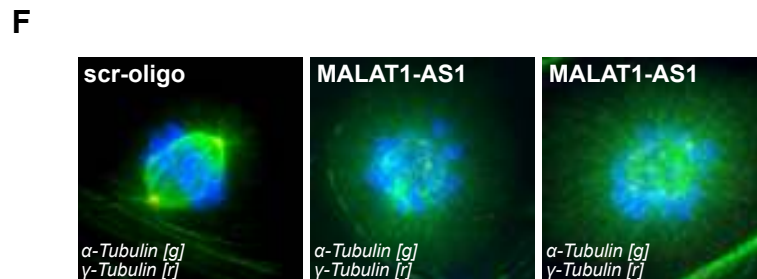

Supplement: Figure S6 — MALAT1 is required for mitotic progression (A) Top significant canonical pathways of the cell cycle genes that are downregulated in MALAT1-depleted fibroblasts. Note that the expression of genes involved in mitotic progression is maximally affected upon MALAT1 depletion. (B) The yellow shaded genes in the pathway analysis diagram are downregulated in MALAT1-depleted fibroblasts, further emphasizing the involvement of MALAT1 in mitotic progression. (C) The relative MALAT1 RNA levels in mitotic (Noco), G1 (Noco 6 hr rel), G1/S synchronized HeLa cells (0 hr rel) and cells that are 12 and 24 hr released in presence of scrambled and MALAT1 antisense oligonucleotides. (D) Flow chart depicting the experimental design. HDFs (WI-38cells) are synchronized in G1/S by serum starvation followed by aphidicolin treatment, followed by MALAT1 depletion and released for 12 and 24 hr to examine the role of MALAT1 in S-phase and mitotic progression. (Ea) Percentage of mitotic cells and cells with broken nuclei in control and MALAT1-depleted HDFs post G1/S (12 and 24 hr) release. (Eb) DAPI-stained nuclei in control and MALAT1-depleted HDFs post 24 hr G1/S release. (F) Immuno-localization of α-tubulin and γ-tubulin in control and MALAT1-depleted WI-38 cells post G1/S (24 hr) release. Note that MALAT1-depleted diploid cells show mitotic segregation defects with monopolar asters. DNA is counterstained with DAPI. Scale bar represents 5 µm. (PDF) [file pgen.1003368.s006.pdf]

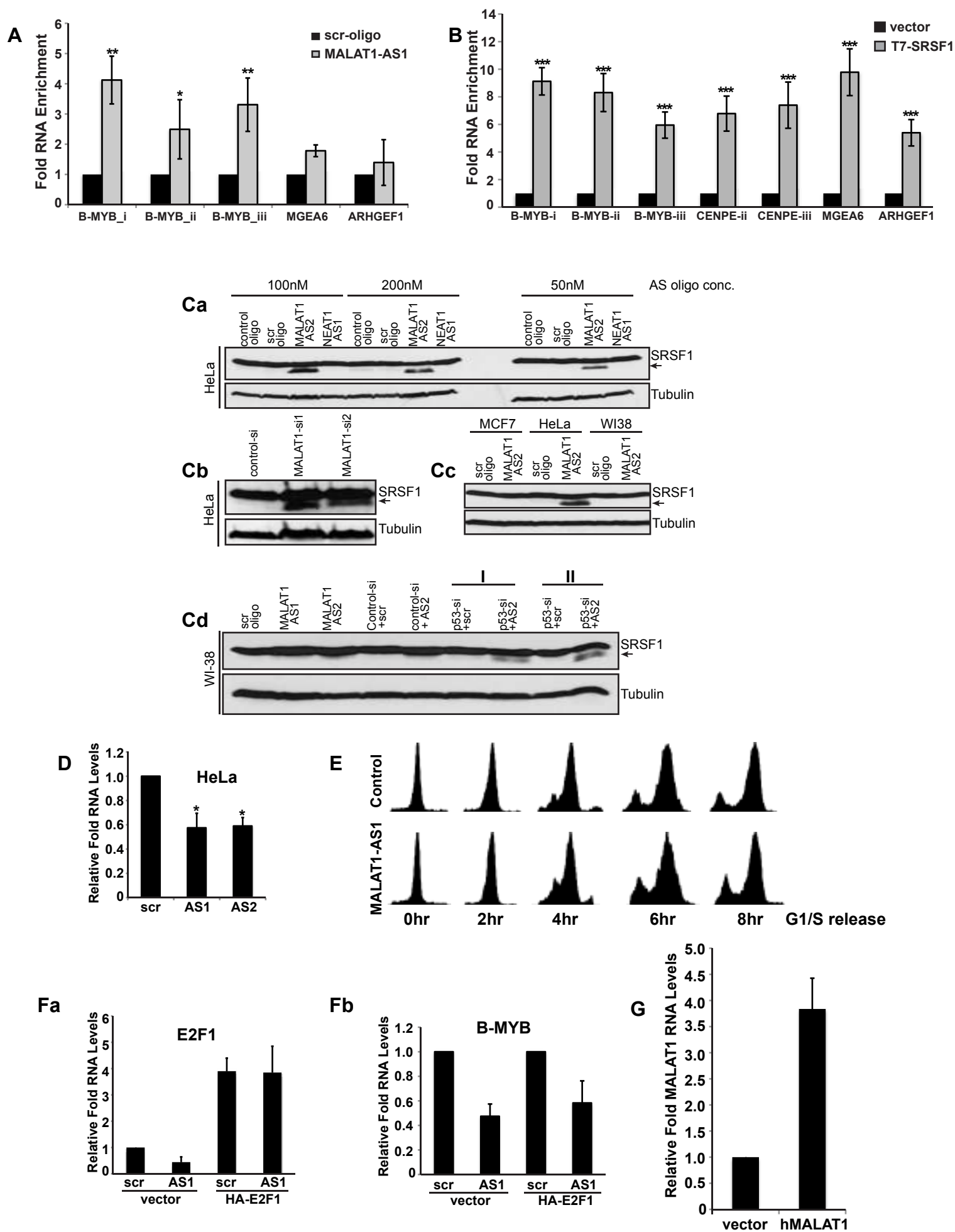

Supplement: Figure S7 — (A) qRT-PCR analyses of SRSF1 RNA-IP samples from control and MALAT1-depleted HeLa cells reveal increased association of SRSF1 to indicated pre-mRNAs. (B) qRT-PCR analyses of T7 RNA-IP samples from control and T7-tagged SRSF1 over expressed HeLa cells reveal increased association of SRSF1 to indicated pre-mRNAs in T7-SRSF1 overexpressed cells. Mean ± SEM, *p<0.05, **p<0.01 and ***p<0.001. (Ca) Immunoblot analysis of SRSF1 in control (control siRNA oligo = luciferase gene siRNA; scr oligo = scrambled oligo against MALAT1 AS2 oligo), MALAT1-AS2) and Neat1-AS1 antisense oligo-treated HeLa cells. The cells are transfected with various concentrations (50, 100 & 200 nM) of siRNA or modified DNA antisense oligos using Lipofectamine RNAimax transfection reagent. The arrow designates the dephosphorylated SRSF1. (Cb) Immunoblot analysis of SRSF1 in control (control si), MALAT1-depleted (using double-stranded siRNAs; si1 & si2) HeLa cell extracts. (Cc) Immunoblot analysis of SRSF1 in control (scr-oligo) and MALAT1-depleted (AS2), MCF7, HeLa and WI-38 cell extracts. Note that the dephosphorylated SRSF1 is present only in MALAT1-depleted HeLa cells. (Cd) Immunoblot analysis of SRSF1 in control (scr-oligo or control siRNA), p53 or MALAT1 or p53 + MALAT1 co-depleted WI-38 cell extracts. Note that p53 and MALAT1 co-depleted WI-38 cells show increased levels of dephosphorylated SRSF1. (D) qRT-PCR using RNA from control and MALAT1-depleted HeLa cells to determine the relative expression of B-MYB. (E) Flow cytometry analyses of control and MALAT1-depleted G1/S synchronized (0 hr) and released (2–8 hr) HeLa cells reveal comparable cell cycle progression. (Fa–b) The relative levels of indicated mRNAs (E2F1 and B-MYB) in control (scr) and MALAT1-depleted (AS1) HDFs that are transiently transfected with vector or E2F1 eukaryotic expression plasmids. (G) The relative levels of MALAT1 are determined by qRT-PCR analyses in RNA from control and MALAT1 overexpressed WI-38 cells. (PDF) [file pgen.1003368.s007.pdf]
