# Supplementary material for: Long Noncoding RNA MALAT1 Controls Cell Cycle Progression by Regulating the Expression of Oncogenic Transcription Factor B-MYB
Source: PLoS Genet. 2013 Mar 21;9(3):e1003368. doi: 10.1371/journal.pgen.1003368 (PMC3605280; doi:10.1371/journal.pgen.1003368)
Supplement: Figure S4 — p53 is a downstream mediator of MALAT1. (A) Co-immunolocalization of α- and γ-tubulin, (B) nucleoporin and lamin A/C in control and MALAT1-depleted HeLa cells. Note the presence of monopolar and highly condensed chromosomes in MALAT1-depleted cells (A). (C) MALAT1 RNA-FISH in control (control-si) and MALAT1-depleted (using MALAT1 siRNAs; si1 & si2) HeLa cells. (Da) MALAT1 RNA-FISH in human MALAT1-depleted HeLa cells that express mouse MALAT1. (Db) Percentage of mouse Malat1 expressing HeLa cells (as observed by MALAT1 RNA-FISH) with normal and broken nuclei upon human MALAT1 depletion. Note that the HeLa cells that express mouse MALAT1 do not show nuclear break down upon depletion of endogenous human MALAT1. (Ea–c) HeLa cells are synchronized in mitosis by nocodazole, incubated with control or MALAT1 antisense oligonucleotides and released for indicated time points to examine the role of MALAT1 in S-phase progression. (Ea) Flow cytometry analyses of control (scr-oligo) and MALAT1-depleted cells (AS1) post-mitotic release. (Eb) The relative MALAT1 RNA levels in control and MALAT1-depleted HeLa cells that are released (12, 15 & 18 hr) post nocodazole treatment. (Ec) BrdU-incorporation assays of control (scr) and MALAT1-depleted cells (AS1) post 12 hr nocodazole release. (F) Table showing the phenotypes (mitosis and cell cycle arrest) observed in several cell lines upon MALAT1 depletion. +++ maximum, ++ medium and + minor changes. ‘M’ designates for mitotic defects and ‘NP’ stands for no obvious phenotype. RKO cells showed cell death upon MALAT1 depletion. (Ga–b) MALAT1 RNA-FISH in control (scr-oligo) and MALAT1-depleted (AS1 & AS2) WI-38 and WI-38-VA13 (WI-38 cells stably expressing SV40-T-antigen). (Ha–c) Relative levels p16Ink4A (Ha), p53 (Hb) and MALAT1 (Hc) RNA is determined by qRT-PCR in WI-38 cells that are transfected with control (scr), p16Ink4A siRNA (p16si), p53 siRNA (p53si), MALAT1 antisense oligos (AS1), or combination of siRNAs with MALAT1 AS1. (I) Q-PCR [file pgen.1003368.s004.pdf]

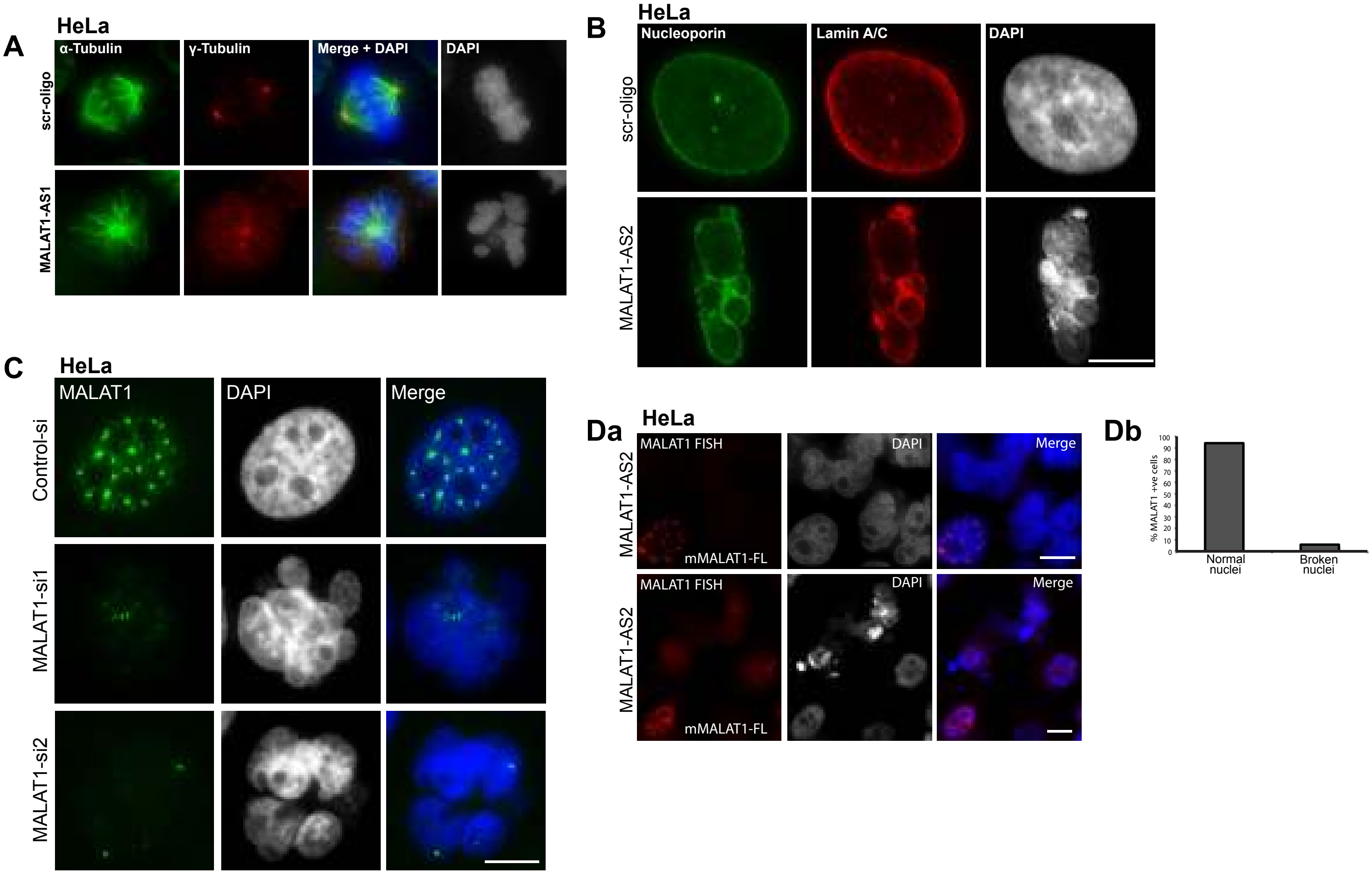

Tripathi et al., Fig-S4

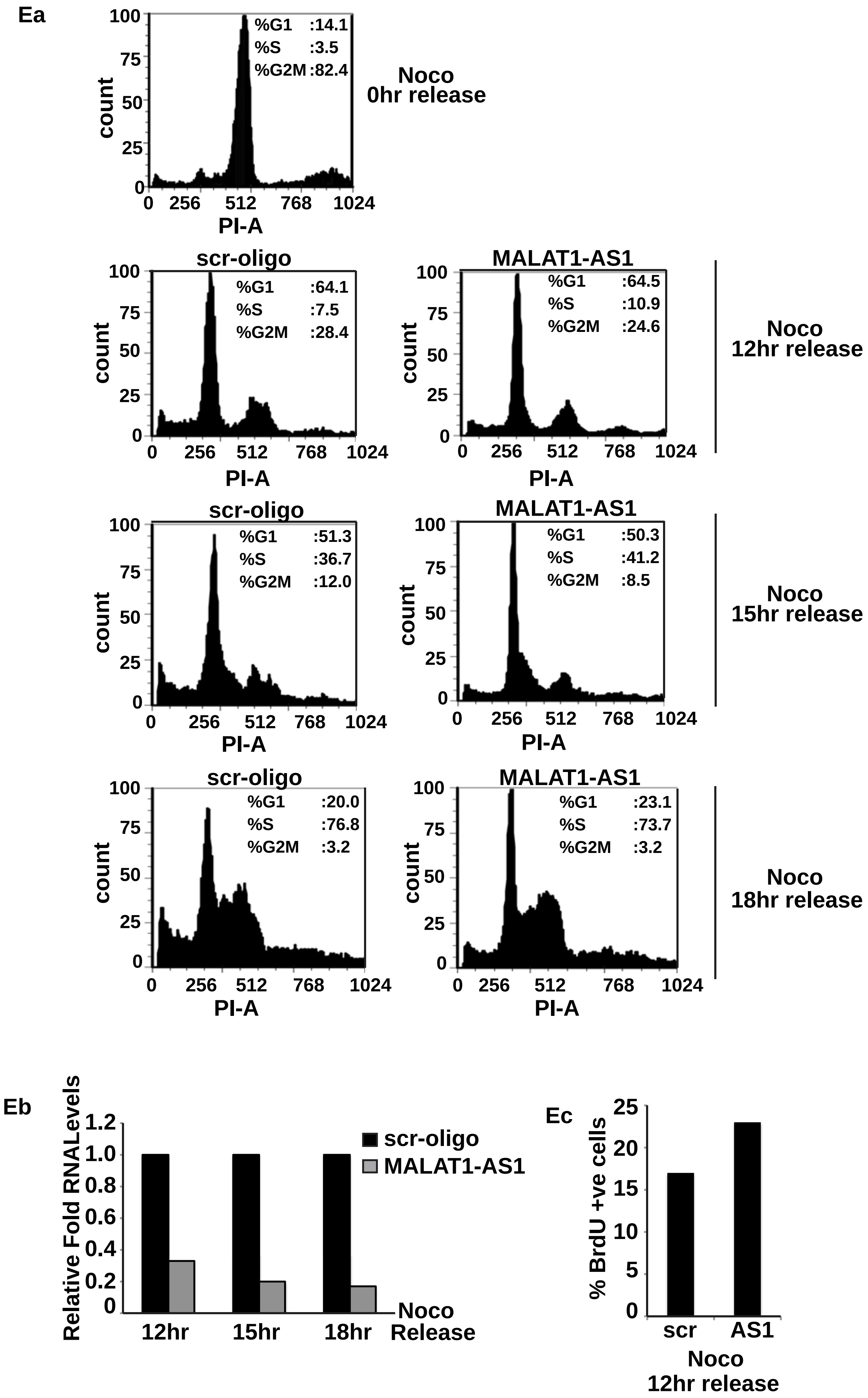

Tripathi et al., Fig-S4

F

Cell lines	Phenotype		
	Aberrant Mitosis	Cell cycle arrest	no obvious phenotype
HeLa	+++	M	
IMR90	+	proliferation defect	
WI-38	+	proliferation defect	
WI-38-VA13	+++	M	
U2OS	+++	M	
HCT116	-	-	cell death
RKO	NP*	NP*	cell death
HepG2	NP	NP	NP
WT-MEF	NP	NP	NP
NIH-3T3	NP	NP	NP

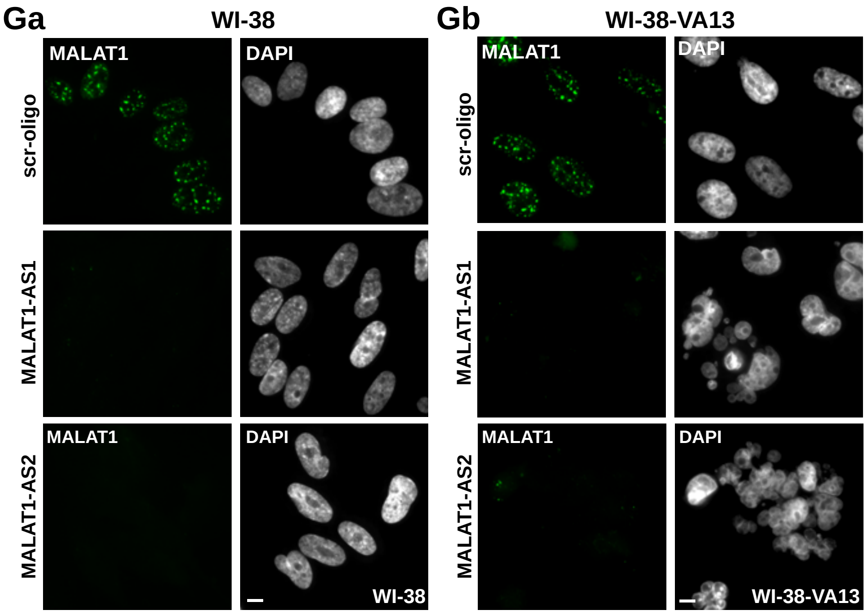

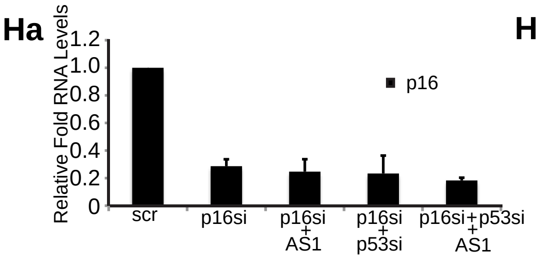

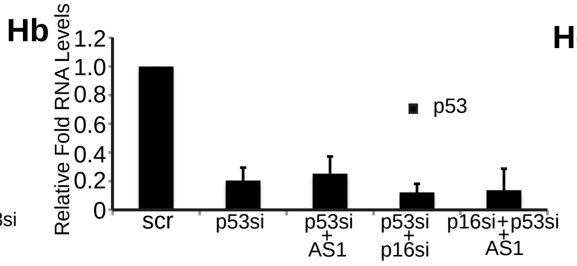

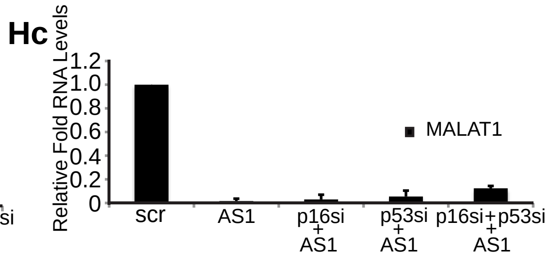

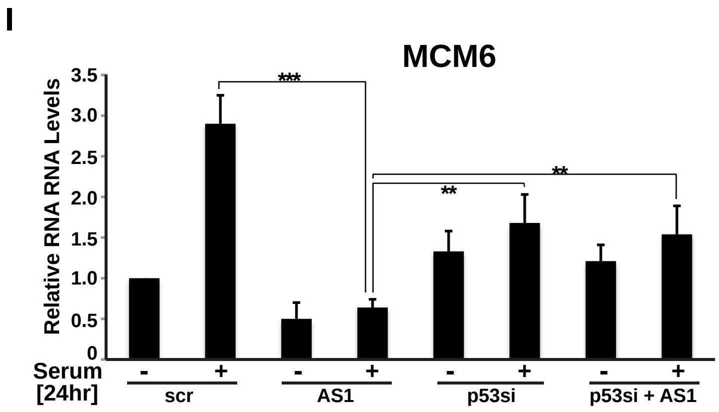

Tripathi et al., Fig-S4
