# Supplementary material for: Long Noncoding RNA MALAT1 Controls Cell Cycle Progression by Regulating the Expression of Oncogenic Transcription Factor B-MYB
Source: PLoS Genet. 2013 Mar 21;9(3):e1003368. doi: 10.1371/journal.pgen.1003368 (PMC3605280; doi:10.1371/journal.pgen.1003368)
Supplement: Protocol S1 — Supporting materials and methods. (DOCX) [file pgen.1003368.s008.docx]

**SUPPLEMENTARY MATERIALS**

**Supplementary methods**

**Plasmid constructs and DNA transfection:**

Plasmid DNAs (500ng-5 μg) were individually transfected into cells using lipofectamine 2000 (Invitrogen, Carlsbad, CA). After transfection, cells were processed for RNA isolation 24 hr post transfection.

**RNA fluorescence *in situ* hybridization (RNA-FISH) and Immunofluorescence staining:**

Fluorescence *in situ* detection of MALAT1 RNA was performed as described earlier [[1](#_ENREF_1)]. Immunolocalization of proteins was performed as described previously [[1](#_ENREF_1),[2](#_ENREF_2)]. Briefly, cells were fixed in 2% formaldehyde for 15 min at room temperature and permeabilized in 0.5% Triton X-100 in PBS for 10min on ice. Cells were incubated with primary and secondary antibodies in a humidified chamber, and finally stained with DAPI and mounted using PPD. For BrdU labeling, cells were incubated in BrdU (10 μM for 20 min) (Sigma, USA), fixed in 2% formaldehyde followed by permeabilization in 0.5% Triton X-100. DNA was denatured with 4N HCl for 30min at room temperature. Further, immunofluorescence analysis was performed using anti-BrdU antibody (Sigma, USA) as mentioned above.

**Immunoblotting:**

Cells were scraped in medium and washed with ice-cold 1X PBS, pH 7.2. Extraction was performed in lysis buffer containing protease and phosphatase inhibitors for 10min on ice. Loading dye was added to the lysate, samples were boiled for 5min and loaded onto a polyacrylamide gel. Western blotting was performed as described previously [[1](#_ENREF_1),[2](#_ENREF_2)].

**Antibodies:**

Antibodies used in the present study include, B-MYB (WB, 1:1000, Millipore), MEK2 (WB, 1:500, BD Pharmingen), p53 (WB, 1:500, Santa Cruz), Cyclin E (WB, 1:1000, Upstate), Lamin B1 (WB 1:1000), Lamin A/C (IF 1:1000) anti-BrdU (IF, 1:1000), Nucleoporin (IF, 1:1000), B”-U2snRNP (WB 1: 250), CENPE (WB, 1:500, Sigma), CBX5 (WB, 1:500), γ-Tubulin (IF, 1:1000; WB, 1:5000; Sigma), α-Tubulin (IF, 1:2000; WB, 1:5000; Sigma) hnRNP-A/B (WB, 1:1000), PCNA (mAbPC10; WB, 1:1500), BUB3 (WB, 1:2000), CDT1 (GP47; WB, 1:200), GMNN (WB, 1:200; Santa Cruz), MCM3 (WB, 1:500), ORC1 (mAbpKS1-40; WB, 1:500), MAD2L1 (WB, 1:500, Sigma), CDC20 (WB, 1:400), PLK1 (WB, 1:50, Santa Cruz), SRSF1 (mAb96 WB, 1:1000), HDM2 (2A10, WB, 1:250) [[3](#_ENREF_3)], phospho-Rb (p-Ser780, WB, 1:500, Cell Signaling, USA) [[4](#_ENREF_4)], γH2AX (clone JBW301, WB, 1:700, Millipore, USA).

**RNA-immunoprecipitation (RNA-IP):**

RNA-IP was performed following the protocol by Sun et al., with minor modifications [[1](#_ENREF_1),[5](#_ENREF_5)]. WI-38 and HeLa cells were treated with control and MALAT1-AS1 oligos, and RNA immunoprecipitation was performed utilizing reversible chemical crosslinking of RNA-protein interactions by formaldehyde followed by immunoprecipitation using SRSF1 (mAb96, Invitrogen, USA) antibody. WI-38 and HeLa cells were transiently transfected with 2 μg T7-tagged plasmid constructs (pCGT-vector, pCGT-SRSF1) and processed for RNA immunoprecipitation using T7 antibody (Novagen, USA) as described above. After IP, extracts were reverse cross-linked, total RNA was extracted using Trizol LS (Invitrogen, USA) and treated with amplification grade DNase I (Invitrogen, USA), and RT-PCR was conducted using random hexamers as per the manufacturer’s instructions (Applied Biosystems, USA). qPCR was performed using specific set of primers (Supplementary table 6).

**Quantitative Real-time PCR (qRT-PCR):**

Total cellular RNA was extracted from the cells using Trizol (Invitrogen, USA) as per manufacturer’s instructions and reverse transcribed into cDNA using Multiscribe Reverse transcriptase and Random hexamers (Applied Biosystems). qRT-PCRs were performed using StepOne Plus system (Applied Biosystems). Transcript levels were quantitated against a standard curve by Real-time RT-PCR using the SYBR Green I fluorogenic dye and data analyzed using the StepOne plus system software. Primer sets showing comparable high efficiencies were used for the analyses.

For the RNA-IP experiment, the qRT-PCR results were analyzed using the comparative Ct method [[6](#_ENREF_6)]. The qRT-PCR levels of the IP samples were normalized against the input of the same samples and calibrated to IgG control. Fold RNA enrichment represents log of RQ value. Histograms represent mean ±SD obtained for three independent experiments. The knockdown or overexpression analyses of MALAT1 RNA and other transcripts were calculated using Q-gene, a Microsoft Excel script package [[7](#_ENREF_7)].

**RT-PCR assays:**

Total cellular RNA was extracted from control and MALAT1 depleted WI-38 cells using Trizol (Invitrogen, USA) and polyA^+^ RNA was isolated using oligo-dT columns (NucleoTrap Midi kit, Clontech, USA) as per the manufacturer’s instructions. One-step RT-PCR was performed using 10 ng of polyA^+^ RNA as template with gene-specific primers (5 μM) along with α-^32^P-dCTP (Perkin Elmer, USA). The total reaction product was run on a 7.5% polyacrylamide gel, dried and exposed to a phosphoimager screen (Storm, Amersham Biosciences). The quantification of the isoform abundance was performed using image analyzer (ImageQuant) and the percentage inclusion of the exons was derived. The specific bands were also eluted and purified from the gel and were sequenced.

**Cell Proliferation Assay:**

Asynchronously growing WI-38 cells were treated with control and MALAT1-AS oligos. 24 hr after the second round of knockdown, cells were trypsinized and equal number of control and MALAT1 depleted cells were plated in 24-welled plates in triplicate. Total number of cells from each well was counted at 0 hr, 24 hr, 48 hr and 72 hr after plating. The experiment was repeated three times and a graph was plotted against the number of cells versus time point to analyze the proliferation rate in control versus MALAT1 depleted cells.

**Cell Synchronization:**

HeLa/U2OS cells were synchronized to mitosis by treatment with 50 ng/ml Nocodazole for 12-16 hr. To synchronize cells in G1, the mitotic cells were washed again with PBS and grown in fresh medium for 5-6 hr. The cells were synchronized to S-phase by double thymidine block and release. Briefly, cells were grown in 2 mM thymidine for 24 hr. After the first block, thymidine was removed and cells were released for 12 hr in fresh medium. Further, 2 mM thymidine was added again for 24 hr to synchronize cells at G1/S. The cells were released in fresh medium for 4 hr to collect them in S phase and 8 hr for G2 phase [[8](#_ENREF_8)].

To study the involvement of MALAT1 in G1/S progression in WI-38 cells, asynchronously growing cells were incubated in medium containing 0.1% serum for 3 days in order to synchronize them to G0 and further incubated with MALAT1-AS oligos on 4^th^ and 5^th^ day in the absence of serum. Cells were then released in medium supplemented with 20% serum and collected at 0 hr, 24 hr and 36 hr of release. The cells were processed directly for flow cytometry, RNA isolation, immunoblotting and BrdU labeling.

To study the involvement of p53 in MALAT1 mediated cell cycle arrest, asynchronously growing HDFs (WI-38) were incubated in medium containing 0.1% serum for 3 days in order to synchronize them to G0 and further incubated with control or p53 siRNA on 4^th^ day followed by control or MALAT1-AS oligos on 5^th^ and 6^th^ day in the absence of serum. Cells were then released in medium supplemented with 20% serum and collected at 0 hr and 24 hr of release. The cells were processed directly for RNA isolation.

To study the involvement of MALAT1 in post-G1/S, WI-38 cells were synchronized to G0 by serum starvation for 3 days. The cells were released in medium with 20% serum and 5 μg/ml Aphidicolin (Sigma) for 24 hr in order to arrest them at G1/S. MALAT1 was depleted using antisense oligos when the cells were already in G1/S. After 24 hr of aphidicolin treatment, cells were released by supplementing with fresh medium and collected at 12 and 24 hr of release. Cells were directly processed for RNA isolation, flow cytometry and immunofluorescence staining.

To study the involvement of MALAT1 in G1/S progression in HeLa cells, mitotic cells were collected by treating the cells with 50 ng/ml nocodazole for 12 hr. Mitotic cells were released in fresh medium containing MALAT1-AS Oligos. Cells were collected at 12hr, 15hrs and 18hrs of release and processed directly for flow cytometry, RNA isolation and BrdU labeling. To study the involvement of MALAT1 in post-G1/S phase, HeLa cells were treated with 50ng/ml nocodazole for 12 hr. Mitotic cells were collected and released in fresh medium containing 5 μg/ml Aphidicolin for 24hr. MALAT1 was depleted using antisense oligos after 15 hr of aphidicolin treatment, when the cells had already arrested at G1/S. At 24 hr, the cells were released in fresh medium and collected at 12 and 24 hr. Cells were directly processed for RNA isolation, flow cytometry and immunofluorescence staining.

**Flow Cytometry:**

Cells were collected and washed in chilled PBS, resuspended in PBS + 1%NGS and fixed in ethanol overnight. Later, cells were washed and resuspended in PBS + 1%NGS with 10 ug/ml RNase A and 120 ug/ml propidium iodide, and further incubated at 37^o^C for 30min. DNA content was measured by flow cytometry.

**SA-β-Gal Staining:**

WI-38 cells were fixed with 0.5% gluteraldehyde (Sigma, USA) solution in PBS for 15min at RT and washed three times with MgCl2/PBS (1mM MgCl_2_ in PBS, pH 6.0) solution. Further, cells were incubated in X-Gal staining solution (1x X-Gal stock + 1x KC in MgCl2/PBS, pH 6.0) at 37^o^C for few hours. [X-Gal stock: 40mg/ml X-Gal (Sigma-Aldrich, USA) in Dimethylformamide. 20xKC: 0.82g K_3_Fe(CN)_6_ + 1.05g K_4_Fe(CN)_6_.3H_2_O + 25 ml MgCl_2_/PBS, pH 6.0]. Once the desired staining was obtained, the cells were washed with water several times and stored in dark. Cells with a positive β-Gal staining were counted and % β-Gal positive cells in control and MALAT1-AS oligo treated cells were estimated.

**References**

1. Tripathi V, Ellis JD, Shen Z, Song DY, Pan Q, et al. (2010) The Nuclear-Retained Noncoding RNA MALAT1 Regulates Alternative Splicing by Modulating SR Splicing Factor Phosphorylation. Mol Cell 39: 925-938.

2. Prasanth KV, Sacco-Bubulya PA, Prasanth SG, Spector DL (2003) Sequential entry of components of the gene expression machinery into daughter nuclei. Mol Biol Cell 14: 1043-1057.

3. Chen J, Marechal V, Levine AJ (1993) Mapping of the p53 and mdm-2 interaction domains. Mol Cell Biol 13: 4107-4114.

4. Guo J, Sheng G, Warner BW (2005) Epidermal growth factor-induced rapid retinoblastoma phosphorylation at Ser780 and Ser795 is mediated by ERK1/2 in small intestine epithelial cells. J Biol Chem 280: 35992-35998.

5. Sun BK, Deaton AM, Lee JT (2006) A transient heterochromatic state in Xist preempts X inactivation choice without RNA stabilization. Mol Cell 21: 617-628.

6. Pfaffl MW (2001) A new mathematical model for relative quantification in real-time RT-PCR. Nucleic Acids Res 29: e45.

7. Muller PY, Janovjak H, Miserez AR, Dobbie Z (2002) Processing of gene expression data generated by quantitative real-time RT-PCR. BioTechniques 32: 1372-1374, 1376, 1378-1379.

8. Shen Z, Sathyan KM, Geng Y, Zheng R, Chakraborty A, et al. (2010) A WD-repeat protein stabilizes ORC binding to chromatin. Mol Cell 40: 99-111.
